# Frailty prediction in heart failure patients with acute infections: the potential role of thiazide diuretics?

**DOI:** 10.1371/journal.pone.0355848

**Published:** 2026-08-12

**Authors:** Tinghui Huang, Shuyi Liu, Siyu Zhang, Xi Song, Ming Xu, Huiling Wu, Jianjun Zou, Yuying Shen

**Affiliations:** 1 Department of General Practice, Nanjing First Hospital, Nanjing Medical University, Nanjing, Jiangsu, China; 2 School of Basic Medicine and Clinical Pharmacy, China Pharmaceutical University, Nanjing, Jiangsu, China; 3 Department of Pharmacy, Nanjing First Hospital, Nanjing Medical University, Nanjing, Jiangsu, China; 4 Department of Pharmacy, Nanjing First Hospital, China Pharmaceutical University, Nanjing, Jiangsu, China; University of Naples Federico II, ITALY

## Abstract

**Background:**

Frailty remains a significant risk factor for adverse health outcomes in hospitalized patients. Few have evaluated frailty risk and its influencing factors in heart failure (HF) patients with acute infections, and previous machine learning models have predominantly overlooked the incorporation of visualization techniques. This study aims to investigate frailty risk factors in this population and develop an interpretable prediction model for frailty.

**Methods:**

This study enrolled 1498 patients hospitalized for HF with acute infections at Nanjing First Hospital in 2023. Participants were randomly divided into training and testing sets at a 7:3 ratio. Potential predictors were screened through univariate analysis and the least absolute shrinkage and selection operator (LASSO) regression. Eight machine learning algorithms were evaluated to determine the optimal predictive model. Model interpretability was enhanced using the SHapley Additive exPlanations (SHAP) method.

**Results:**

Frailty was prevalent in 80.3% of the cohort. Key predictors included the use of thiazide diuretics, serum albumin, estimated glomerular filtration rate (eGFR), lymphocyte percentage, mean corpuscular hemoglobin concentration (MCHC), capacity for action, age, left ventricular ejection fraction (LVEF), New York Heart Association (NYHA) functional class, history of cerebral infarction, and smoking. Comparative analysis of the eight models revealed that eXtreme Gradient Boosting (XGBoost) achieved superior performance, with the highest area under the receiver operating characteristic curve (AUROC: 0.872) and precision-recall curve (AUPRC: 0.969).

**Conclusions:**

This study identified the use of thiazide diuretics as an independent predictor associated with lower frailty probability. We developed an online calculator as a proof-of-concept tool to demonstrate the potential application of the predictive model and facilitate real-time risk estimation.

## Introduction

Frailty, a multidimensional geriatric syndrome closely associated with aging, manifests as an excessive decline in reserve and function across multiple physiological systems, leading to diminished responsiveness to minor stressors [1]. It is highly prevalent among hospitalized patients and significantly increases the risk of adverse health outcomes, including hospitalization, falls, disability, and mortality [2,3]. It is noteworthy that heart failure (HF), similar to frailty, represents a complex clinical syndrome with a persistently increasing hospitalization prevalence, which has become a significant global public health concern [4–6].

However, previous research on frailty has primarily focused on stable chronic disease populations [7]. However, frailty-related risk factors in patients with coexisting acute and chronic conditions—a common yet understudied clinical scenario—remain largely unexplored. In HF with acute infection, the overlapping pathophysiology of fluid retention (HF) and fluid exudation (infection) may synergistically exacerbate fluid overload, manifesting as bilateral lower extremity edema and secondary muscle strength decline, thereby increasing the risk of prolonged bed rest and adverse outcomes [8]. These complications not only worsen prognosis but also impose substantial challenges to clinical management [9,10].

Moreover, existing frailty prediction models, whether based on conventional regression or machine learning approaches, have notable limitations. Logistic regression-based nomograms, while achieving acceptable discrimination (e.g., AUCs of 0.912 and 0.881 in diabetic patients [11]; AUC of 0.847 in elderly coronary heart disease patients [12]), often suffer from limited practical utility or small sample sizes. Machine learning-based models, such as an XGBoost model for chronic obstructive pulmonary disease (COPD) patients using China Health and Retirement Longitudinal Study (CHARLS) data (test AUC 0.942) [13], have demonstrated superior performance but frequently lack objective laboratory parameters. Moreover, most existing models overlook model interpretability—a critical barrier to clinical adoption, as clinicians require intuitive understanding of how individual predictors contribute to frailty risk at the patient level. SHAP (SHapley Additive exPlanations), a game theory-based approach, quantifies each feature’s marginal contribution to individual predictions, effectively bridging the gap between predictive accuracy and clinical transparency [14,15].

To solve these disadvantages, this study aimed to investigate frailty-associated risk factors in patients with concurrent HF and acute infections, and to develop a frailty prediction model utilizing multiple machine learning algorithms. Additionally, a clinically accessible online calculator was developed and deployed to accurately identify high-risk individuals and tailor appropriate interventions based on individual risk factors. Ultimately, this approach sought to reduce the incidence of frailty or even reverse its progression in affected patients.

## Methods

### Design and participants

This study collected clinical data from patients with HF complicated by acute infections at Nanjing First Hospital between January 1 and December 31, 2023, and employed various machine learning algorithms to predict frailty in this patient population. The data used for this study were accessed for research purposes on 15/04/2025 from the hospital’s electronic medical record system. The inclusion criteria were as follows: (1) age ≥ 65 years; (2) diagnosed with HF according to the Chinese Guidelines for the Diagnosis and Treatment of Heart Failure 2024 [16]; (3) evidence of acute infections, including fever, tachycardia, elevated inflammatory markers, or imaging findings suggestive of infection. The exclusion criteria were as follows: (1) incomplete medical records; (2) uninfected patients; (3) comorbidities such as acute myocardial infarction, advanced malignancy, psychiatric disorders, or severe trauma; (4) New York Heart Association (NYHA) functional class I. This study was approved by the Ethics Committee of Nanjing First Hospital (Approval No. KY20250120-KS-03). As this was a retrospective study, the Ethics Committee waived the requirement for informed consent. This study was conducted by the ethical standards outlined in the Declaration of Helsinki.

### Frailty status assessment

All patients were assessed for frailty using the Clinical Frailty Scale (CFS), with scores ranging from 1 (very fit) to 9 (terminally ill). According to the CFS scoring criteria, a score≤4 indicates a non-frail state, while a score≥5 is classified as frail [17]. The CFS assessment for all patients was performed by a nurse who received standardized training. To ensure consistency and standardization in the assessments, all participating nurses underwent specialized training before data collection. The training covered the theoretical background of the CFS, detailed definitions for each level of the scale, and specific assessment procedures. A senior geriatrician conducted the entire training process, assessment supervision, and quality control. The assessments strictly adhered to the internationally recognized CFS scoring form, which has been validated in Chinese populations [18]. Furthermore, an outcome-blinding design was implemented to mitigate potential bias. Nurses conducting CFS assessments were excluded from clinical decision-making for the patients. Assessments were finalized upon resolution of the acute infection phase and at the time of hospital discharge, with all results systematically documented in the electronic medical records. The CFS includes the Activities of Daily Living (ADL) scale (eating, bathing, grooming (toothbrushing, face washing, shaving, hair combing), dressing (fastening shoes, buttoning clothes), bowel control, bladder control and toilet use, transfers (bed-to-chair mobility), ambulation (walking 45 meters on level ground), stair climbing) and the Instrumental Activities of Daily Living (IADL) scale (telephone usage, shopping, meal preparation, housekeeping, laundry, transportation, medication management, financial handling).

### Clinical data collection

All clinical data were extracted from the electronic medical record system, including: (1) demographic characteristics: age, sex, literacy, marital status, Body Mass Index (BMI), hospital days, capacity for action, smoking, drinking, and NYHA functional class; (2) medication use: angiotensin receptor neprilysin inhibitor (ARNI), angiotensin-converting enzyme inhibitor (ACEI) angiotensin receptor blocker (ARB), sodium-glucose cotransporter protein-2 (SGLT-2) inhibitors, beta receptor blockers, mineralocorticoid receptor antagonist (MRA), calcium channel blocker (CCB), loop diuretics, thiazide diuretics, nitrates, statins, antiplatelet drugs, anticoagulants, cardiac glycosides, soluble guanylate cyclase (sGC) stimulators; (3) comorbidities: hypertension, diabetes mellitus, hyperlipidemia, coronary heart disease, atrial fibrillation, fatty liver disease, cirrhosis, COPD, chronic cor pulmonale, renal insufficiency, osteoporosis, cerebral infarction, malignant tumor; (4) infection characteristics: pulmonary infection, urinary tract infection, abdominal infection, skin and soft tissue infection, bloodstream infection, sepsis, septic shock; (5) admission vital signs: first recorded blood pressure (systolic blood pressure, diastolic blood pressure) and heart rate; (6) biomarkers: white blood cell, lymphocyte percentage, neutrophil percentage, red blood cell, hemoglobin, mean corpuscular hemoglobin concentration (MCHC), platelet, D-dimer, alanine aminotransferase, aspartate aminotransferase, serum albumin, total bilirubin, urea, creatinine, uric acid, serum potassium, serum sodium, total cholesterol, triglycerides, high-density lipoprotein, low-density lipoprotein, estimated glomerular filtration rate (eGFR), interleukin-6, procalcitonin, c-reactive protein, B-type natriuretic peptide (BNP), N-terminal pro-BNP (NT-proBNP), elevated natriuretic peptide levels were defined as BNP>100pgmL or NT-proBNP>300pgmL [19]; (7) echocardiography: left ventricular ejection fraction (LVEF), aortic diameter (AOD), left atrial diameter (LAD), interventricular septum diameter (IVSD), left ventricular end diastolic diameter (LVEDD), left ventricular posterior wall diameter (LVPWD), left ventricular end systolic diameter (LVESD).

### Ethics statement

This study was approved by the Ethics Committee of Nanjing First Hospital (Approval No. KY20250120-KS-03). As this was a retrospective study, the Ethics Committee waived the requirement for informed consent. This study was conducted by the ethical standards outlined in the Declaration of Helsinki.

### Sample size justification

Prior to model development, we estimated the minimum required sample size using the events per variable (EPV) criterion [20]. Based on a review of similar prediction models in the literature [21–23] and our preliminary data, we anticipated that the final model might contain up to 15 predictors. Adhering to the EPV criterion with a threshold of at least 20 EPV, we required a minimum of 300 outcome events. In our training set, we observed 834 frail cases, yielding an actual EPV of 55.6 (834/15), which met our pre-specified requirement.

### Data preprocessing

All analyses began with a rigorous cleaning protocol applied to the raw dataset. First, duplicate records were screened using unique participant identifiers; none were found. Next, potential outliers in variables were evaluated through manual screening of the original data. Any values deemed clinically unusual were cross-verified against original source records and discussed with relevant clinical staff to assess their validity. No observations were excluded based on this review process. Of the 81 variables included in the study, six contained missing values, each with a missingness rate below 18% (S1 Table). Missing data were imputed using the K-nearest neighbors (KNN) algorithm after splitting the dataset into training and testing sets. The optimal k value (k = 5) was determined by minimizing the root mean square error (RMSE) of imputation performance, assessed via internal cross-validation within the training set. The KNN imputation model was fitted exclusively on the training set, and the learned imputation strategy was subsequently applied to the testing set. The KNN algorithm estimates missing entries based on Euclidean distances in the feature space among the k most similar observations [24]. This approach preserves underlying data structure and inter-variable relationships while avoiding listwise deletion. KNN imputation was implemented using the KNNImputer class from the scikit-learn library (version 1.2.2).

### Data analysis

The normality of continuous variables was assessed by the Shapiro-Wilk test: normally distributed variables were presented as mean ± SD, and group comparisons were made using independent samples t-tests; non-normally distributed variables were summarized as median (interquartile range) [M (IQR)], and the Mann-Whitney U test was used for group comparisons. Categorical variables were described as frequencies (percentages) [n (%)], and the chi-square test or Fisher’s exact test was selected for analysis of differences between groups according to sample size characteristics. All statistical tests were two-tailed, and P  <  0.05 was considered statistically significant.

### Model development

All analyses described in this section were performed in R (v4.3.3). The dataset was randomly split(set.seed(42)) into training and testing sets in a 7:3 ratio. The training set was used for variable selection and model development, while the testing set evaluated model performance. Continuous variables were standardized using Z-score normalization based on the mean and standard deviation calculated from the training dataset, and the same scaling parameters were subsequently applied to the testing dataset. Categorical variables were encoded via one-hot encoding [25,26]. All feature selection procedures were performed exclusively within the training dataset. Variables significantly associated with frailty in univariate analysis (p < 0.05) were retained for subsequent LASSO regression. Variables significantly associated with frailty (p < 0.05) in univariate analysis were retained for further analysis. The descriptive statistics and univariate comparisons were generated using the tableone package (version 0.13.2). Significant variables were further analyzed using least absolute shrinkage and selection operator (LASSO) regression, implemented in R with the glmnet package (version 4.1.8). LASSO applies L1 regularization to perform automatic feature selection by shrinking the coefficients of less informative predictors toward zero. The regularization parameter λ was tuned via 10-fold cross-validation on the training set, and the optimal λ was selected using the “1-standard-error” rule to favor a more parsimonious model while maintaining predictive performance [27]. Only variables with non-zero coefficients at the chosen λ were retained for subsequent modeling. To assess multicollinearity, variables with a variance inflation factor (VIF) ≥ 5 were excluded to ensure feature independence [28]. VIF was calculated in R using the car package (version 3.1.2).

Given the high frailty prevalence (80.3%), all models that natively support class weighting—including logistic regression (LR), random forest (RF), eXtreme Gradient Boosting (XGBoost), light gradient boosting machines (LightGBM), support vector machine (SVM), categorical boosting (CatBoost) —were trained with automatic adjustment (class weight = ’balanced’ or equivalent) to reduce bias toward the majority class. Naive Bayes (NB) and multilayer perceptron (MLP), which do not support this functionality, were included as unadjusted benchmark models. Meanwhile, we prioritized AUROC and AUPRC as core evaluation metrics, as these indicators are more robust to skewed class distributions compared to raw accuracy, ensuring reliable assessment of the model’s discriminative power across both frail and non-frail subgroups.

All analyses described in this section were performed in Python (v3.11.7). We performed 10-fold cross-validation on the training set (with random_state = 1) to tune hyperparameters, ensuring reproducible fold splits. In each fold, the model was trained on 9 folds and validated on the remaining fold, with all 10 folds used once as the validation set. For each candidate hyperparameter configuration, we computed the mean ROC-AUC across the 10 validation folds using scikit-learn (v1.2.2) and selected the configuration with the highest average performance. The independent testing set was not involved in hyperparameter optimization or model selection. The final model was then retrained on the full training set using these optimal hyperparameters, with random_state = 1 to ensure reproducibility, and its performance was subsequently evaluated on the independent testing set. The final model was evaluated through: (1) performance metrics on the testing set, including the area under the receiver operating characteristic curve (AUROC), area under the precision-recall curve (AUPRC), sensitivity, specificity, positive predictive value(PPV), negative predictive value(NPV), and F1-score, with 95% confidence intervals derived from 2,000 bootstrap (np.random.seed(42)) replicates, implemented using NumPy (v1.26.4) and scikit-learn (v1.2.2); (2) calibration curves to evaluate the agreement between predicted and observed probabilities, complemented by Brier scores quantifying overall calibration error, both computed with scikit-learn (v1.2.2) and visualized using Matplotlib (v3.8.0); (3) decision curve analysis (DCA) to quantify net clinical benefit, including standardized net benefit rates and optimal risk thresholds performed using the dcurves package (v1.1.7). To interpret the model, SHAP were applied to quantify feature contributions [29], implemented via the shap library (v0.47.0) with plots rendered using Matplotlib (v3.8.0). To facilitate demonstration of the predictive model, an interactive web application was developed using Streamlit (v1.30.0) to visualize predictions.

## Result

### Sample characteristics

**Fig 1:**
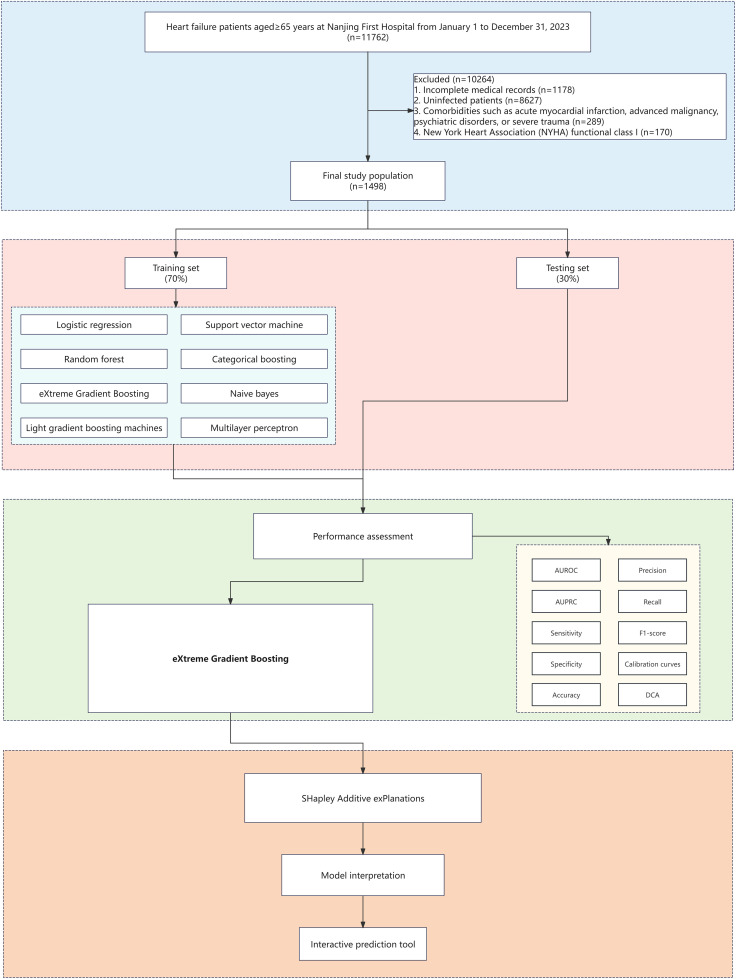
The schematic of the study workflow. Abbreviations: AUROC, Area Under The Receiver Operating Characteristic Curve; AUPRC, Area Under Precision-Recall Curve; DCA, Decision Curve Analysis.

This study initially screened 11762 hospitalized patients, excluding 10264 cases that met the exclusion criteria. Ultimately, 1498 patients were included in the analysis, with 1203 (80.3%) diagnosed with frailty. The median age was 77 years, with 59.8% males and 40.2% females. The mean hospital stay was 10.5 days. Baseline characteristics of the full cohort are presented in S2 Table. There were no significant differences in variables between the training and testing sets (S3 Table). The schematic of the study workflow is detailed in Fig 1.

### Variables selection

This study initially considered 81 clinically relevant variables. To identify variables potentially associated with frailty, we first conducted univariate analyses. For continuous variables, group differences were assessed using the independent samples t-test or Mann-Whitney U test, depending on distributional normality. For categorical variables, the chi-square test or Fisher’s exact test was applied as appropriate. Variables showing significant associations in univariate analyses (p < 0.05) were retained as candidate predictors for subsequent LASSO-based selection, resulting in 37 candidate predictors. (Table 1).

**Table 1:** Demographics and potential risk factors of patients in the training set.

Variables	Overall (n = 1048)	No Frailty(n = 214)	Frailty (n = 834)	p
Sex, n (%)				0.004*
Female	425 (40.6)	68 (31.8)	357 (42.8)	
Male	623 (59.4)	146 (68.2)	477 (57.2)	
Marital Status, n (%)				<0.001*
Unmarried	3 (0.3)	0 (0.0)	3 (0.4)	
Divorced And Widowed	79 (7.5)	2 (0.9)	77 (9.2)	
Married	966 (92.2)	212 (99.1)	754 (90.4)	
Literacy, n (%)				0.126
Illiteracy	172 (16.4)	24 (11.2)	148 (17.7)	
Primary School	329 (31.4)	69 (32.2)	260 (31.2)	
Junior High School	284 (27.1)	67 (31.3)	217 (26.0)	
Senior High School	166 (15.8)	31 (14.5)	135 (16.2)	
College Degree Or Above	97 (9.3)	23 (10.7)	74 (8.9)	
Capacity For Action, n (%)				<0.001*
Bedridden	323 (30.8)	2 (0.9)	321 (38.5)	
Wheelchair-Dependent	88 (8.4)	0 (0.0)	88 (10.6)	
Ambulatory	637 (60.8)	212 (99.1)	425 (51.0)	
Smoking, n (%)				0.008*
No	727 (69.4)	165 (77.1)	562 (67.4)	
Yes	321 (30.6)	49 (22.9)	272 (32.6)	
Drinking, n (%)				0.022
No	979 (93.4)	192 (89.7)	787 (94.4)	
Yes	69 (6.6)	22 (10.3)	47 (5.6)	
NYHA Functional Class, n (%)				<0.001*
Ⅱ	412 (39.3)	113 (52.8)	299 (35.9)	
Ⅲ	446 (42.6)	86 (40.2)	360 (43.2)	
Ⅳ	190 (18.1)	15 (7.0)	175 (21.0)	
Medicine, n (%)				0.266
＜5	423 (40.4)	94 (43.9)	329 (39.4)	
≥5	625 (59.6)	120 (56.1)	505 (60.6)	
ARNI, n (%)				0.218
No	817 (78.0)	174 (81.3)	643 (77.1)	
Yes	231 (22.0)	40 (18.7)	191 (22.9)	
SGLT-2 Inhibitors, n (%)				0.402
No	838 (80.0)	176 (82.2)	662 (79.4)	
Yes	210 (20.0)	38 (17.8)	172 (20.6)	
Beta Receptor Blockers, n (%)				0.291
No	657 (62.7)	127 (59.3)	530 (63.5)	
Yes	391 (37.3)	87 (40.7)	304 (36.5)	
MRA, n (%)				0.008*
No	577 (55.1)	100 (46.7)	477 (57.2)	
Yes	471 (44.9)	114 (53.3)	357 (42.8)	
ACEI/ARB, n (%)				0.475
No	923 (88.1)	192 (89.7)	731 (87.6)	
Yes	125 (11.9)	22 (10.3)	103 (12.4)	
CCB, n (%)				0.032*
No	759 (72.4)	168 (78.5)	591 (70.9)	
Yes	289 (27.6)	46 (21.5)	243 (29.1)	
Loop Diuretics, n (%)				0.702
No	632 (60.3)	132 (61.7)	500 (60.0)	
Yes	416 (39.7)	82 (38.3)	334 (40.0)	
Thiazide Diuretics, n (%)				<0.001*
No	973 (92.8)	178 (83.2)	795 (95.3)	
Yes	75 (7.2)	36 (16.8)	39 (4.7)	
Nitrates, n (%)				0.232
No	939 (89.6)	197 (92.1)	742 (89.0)	
Yes	109 (10.4)	17 (7.9)	92 (11.0)	
Statins, n (%)				0.176
No	542 (51.7)	120 (56.1)	422 (50.6)	
Yes	506 (48.3)	94 (43.9)	412 (49.4)	
Antiplatelet Drugs, n (%)				0.116
No	591 (56.4)	110 (51.4)	481 (57.7)	
Yes	457 (43.6)	104 (48.6)	353 (42.3)	
Anticoagulants, n (%)				0.106
No	740 (70.6)	141 (65.9)	599 (71.8)	
Yes	308 (29.4)	73 (34.1)	235 (28.2)	
Cardiac Glycosides, n (%)				0.019*
No	979 (93.4)	208 (97.2)	771 (92.4)	
Yes	69 (6.6)	6 (2.8)	63 (7.6)	
sGC Stimulators, n (%)				0.669
No	1038 (99.0)	213 (99.5)	825 (98.9)	
Yes	10 (1.0)	1 (0.5)	9 (1.1)	
Hypertension, n (%)				0.833
No	327 (31.2)	65 (30.4)	262 (31.4)	
Yes	721 (68.8)	149 (69.6)	572 (68.6)	
Diabetes Mellitus, n (%)				0.101
No	662 (63.2)	146 (68.2)	516 (61.9)	
Yes	386 (36.8)	68 (31.8)	318 (38.1)	
Hyperlipidemia, n (%)				0.914
No	1005 (95.9)	206 (96.3)	799 (95.8)	
Yes	43 (4.1)	8 (3.7)	35 (4.2)	
Coronary Heart Disease, n (%)				0.692
No	495 (47.2)	98 (45.8)	397 (47.6)	
Yes	553 (52.8)	116 (54.2)	437 (52.4)	
Atrial Fibrillation, n (%)				0.084
No	751 (71.7)	164 (76.6)	587 (70.4)	
Yes	297 (28.3)	50 (23.4)	247 (29.6)	
Fatty Liver Disease, n (%)				0.002*
No	1022 (97.5)	202 (94.4)	820 (98.3)	
Yes	26 (2.5)	12 (5.6)	14 (1.7)	
Cirrhosis, n (%)				0.78
No	1042 (99.4)	212 (99.1)	830 (99.5)	
Yes	6 (0.6)	2 (0.9)	4 (0.5)	
COPD, n (%)				0.499
No	929 (88.6)	193 (90.2)	736 (88.2)	
Yes	119 (11.4)	21 (9.8)	98 (11.8)	
Chronic Cor Pulmonale, n (%)				0.095
No	1025 (97.8)	213 (99.5)	812 (97.4)	
Yes	23 (2.2)	1 (0.5)	22 (2.6)	
Renal Insufficiency, n (%)				<0.001*
No	776 (74.0)	190 (88.8)	586 (70.3)	
Yes	272 (26.0)	24 (11.2)	248 (29.7)	
Osteoporosis, n (%)				0.494
No	1036 (98.9)	213 (99.5)	823 (98.7)	
Yes	12 (1.1)	1 (0.5)	11 (1.3)	
Cerebral Infarction, n (%)				<0.001*
No	791 (75.5)	188 (87.9)	603 (72.3)	
Yes	257 (24.5)	26 (12.1)	231 (27.7)	
Malignant Tumor, n (%)				0.901
No	970 (92.6)	199 (93.0)	771 (92.4)	
Yes	78 (7.4)	15 (7.0)	63 (7.6)	
Pulmonary Infection, n (%)				0.88
No	59（5.6）	13（6.1）	46（5.5）	
Yes	989 (94.4)	201 (93.9)	788 (94.5)	
Urinary Tract Infection, n (%)				0.117
No	994 (94.8)	208 (97.2)	786 (94.2)	
Yes	54 (5.2)	6 (2.8)	48 (5.8)	
Abdominal Infection, n (%)				0.844
No	1019 (97.2)	209 (97.7)	810 (97.1)	
Yes	29 (2.8)	5 (2.3)	24 (2.9)	
Skin And Soft Tissue Infection, n (%)				0.199
No	1030 (98.3)	213 (99.5)	817 (98.0)	
Yes	18 (1.7)	1 (0.5)	17 (2.0)	
Bloodstream Infection, n (%)				0.428
No	1028 (98.1)	208 (97.2)	820 (98.3)	
Yes	20 (1.9)	6 (2.8)	14 (1.7)	
Sepsis, n (%)				0.063
No	1015 (96.9)	212 (99.1)	803 (96.3)	
Yes	33 (3.1)	2 (0.9)	31 (3.7)	
Septic Shock, n (%)				1
No	1033 (98.6)	211 (98.6)	822 (98.6)	
Yes	15 (1.4)	3 (1.4)	12 (1.4)	
BNP, n (%)				<0.001*
No	261 (24.9)	86 (40.2)	175 (21.0)	
Yes	787 (75.1)	128 (59.8)	659 (79.0)	
Systolic Blood Pressure, mmHg	130.00 [119.00, 143.00]	130.00 [120.00, 141.75]	130.00 [118.00, 143.75]	0.946
Diastolic Blood Pressure, mmHg	76.00 [68.00, 83.00]	76.00 [70.00, 81.00]	76.00 [68.00, 83.75]	0.83
Heart Rate, bpm	80.00 [72.00, 93.00]	80.00 [70.00, 88.75]	81.50 [73.00, 95.00]	0.001*
White Blood Cell, 10^9^/L	6.86 [5.27, 9.20]	6.20 [5.01, 8.32]	7.08 [5.32, 9.42]	0.002*
Lymphocyte Percentage, %	16.10 [9.50, 23.30]	21.95 [15.00, 29.48]	14.55 [8.62, 21.98]	<0.001*
Neutrophil Percentage, %	74.60 [65.18, 83.30]	68.30 [59.12, 76.65]	76.40 [66.95, 84.70]	<0.001*
Red Blood Cell, 10^9^/L	4.01 [3.52, 4.47]	4.28 [3.88, 4.57]	3.92 [3.45, 4.41]	<0.001*
Hemoglobin, g/L	121.00 [105.00, 135.00]	130.00 [118.00, 140.75]	119.00 [102.00, 133.00]	<0.001*
MCHC, g/L	326.00 [320.00, 333.00]	330.00 [324.00, 334.75]	326.00 [318.00, 332.00]	<0.001*
Platelet, 10^9^/L	174.00 [129.00, 228.00]	173.00 [134.00, 224.50]	174.00 [128.00, 228.00]	0.704
D-Dimer, ug/mL	0.88 [0.42, 2.11]	0.46 [0.26, 0.91]	1.08 [0.53, 2.43]	<0.001*
Alanine Aminotransferase, u/L	17.05 [12.00, 27.08]	18.25 [13.00, 26.00]	17.00 [11.00, 27.73]	0.056
Aspartate Aminotransferase, u/L	22.00 [17.00, 32.00]	20.00 [17.00, 28.00]	23.00 [17.00, 33.00]	0.067
Serum Albumin, g/L	35.35 [31.50, 38.50]	38.20 [35.00, 39.90]	34.60 [30.89, 37.90]	<0.001*
Total Bilirubin, umol/L	11.16 [7.60, 16.00]	11.55 [8.03, 16.04]	11.00 [7.43, 16.00]	0.355
Urea, mmol/L, mmol/L	7.35 [5.48, 10.73]	6.10 [5.07, 7.90]	7.74 [5.67, 11.48]	<0.001*
Creatinine, umol/L	84.45 [66.38, 120.43]	73.90 [62.74, 90.30]	89.05 [67.81, 128.90]	<0.001*
Uric Acid, umol/L	330.00 [246.65, 432.50]	316.36 [240.75, 394.65]	338.50 [247.50, 446.45]	0.035*
Serum Potassium, mmol/L	3.84 [3.55, 4.19]	3.79 [3.52, 4.08]	3.86 [3.55, 4.22]	0.038*
Serum Sodium, mmol/L	139.50 [136.90, 141.80]	139.55 [137.80, 141.50]	139.50 [136.70, 141.90]	0.469
Total Cholesterol, mmol/L	3.56 [2.94, 4.32]	3.86 [3.22, 4.48]	3.49 [2.87, 4.28]	<0.001*
Triglycerides, mmol/L	1.02 [0.78, 1.42]	1.10 [0.84, 1.49]	1.01 [0.77, 1.41]	0.016*
High-Density Lipoprotein, mmol/L	0.93 [0.76, 1.12]	0.97 [0.82, 1.13]	0.92 [0.74, 1.12]	0.014*
Low-Density Lipoprotein, mmol/L	1.94 [1.45, 2.54]	2.21 [1.68, 2.64]	1.88 [1.42, 2.52]	<0.001*
eGFR, ml/min	68.32 [43.56, 85.26]	84.97 [66.05, 91.14]	62.96 [40.16, 82.04]	<0.001*
Interleukin-6, pg/mL	15.02 [5.03, 50.56]	12.42 [3.50, 56.88]	16.50 [5.69, 46.87]	0.08
Procalcitonin, pg/mL	0.10 [0.05, 0.35]	0.08 [0.04, 0.19]	0.11 [0.05, 0.42]	0.001*
C-Reactive Protein, mg/L	17.81 [4.86, 59.95]	7.69 [2.52, 40.55]	20.19 [6.19, 63.43]	<0.001*
LVEF, %	61.00 [49.75, 64.00]	63.00 [58.25, 65.00]	61.00 [48.00, 63.00]	<0.001*
AOD, mm	34.00 [31.00, 36.00]	34.00 [31.00, 37.00]	33.00 [31.00, 36.00]	0.494
LAD, mm	44.00 [40.00, 49.00]	44.00 [38.25, 49.00]	44.00 [40.00, 49.00]	0.455
IVSD, mm	10.00 [9.00, 11.00]	10.00 [10.00, 11.00]	10.00 [9.00, 11.00]	0.2
LVEDD, mm	50.00 [46.00, 55.00]	50.00 [47.00, 55.00]	50.00 [46.00, 55.00]	0.365
LVPWD, mm	9.00 [9.00, 10.00]	10.00 [9.00, 10.00]	9.00 [9.00, 10.00]	0.175
LVESD, mm	33.00 [30.00, 40.00]	33.00 [30.00, 37.00]	33.00 [30.00, 40.38]	0.464
Age, year	77.00 [72.00, 84.00]	72.00 [68.00, 75.75]	79.00 [74.00, 85.00]	<0.001*
Hospital Days, day	11.00 [8.00, 16.00]	11.00 [7.00, 18.00]	10.00 [8.00, 15.00]	0.209
BMI, kg/m^2^	23.44 [21.08, 25.78]	24.03 [21.99, 25.91]	23.23 [20.81, 25.75]	0.006*

Data are presented as n (%) or median (IQR). **Abbreviations**: NYHA Functional Class, New York Heart Association Functional Class; ARNI, Angiotensin Receptor Neprilysin Inhibitor; SGLT-2 Inhibitors, Sodium-Glucose Cotransporter Protein-2 Inhibitors; MRA, Mineralocorticoid Receptor Antagonist; ACEI, Angiotensin-Converting Enzyme Inhibitor; ARB, Angiotensin Receptor Blocker; CCB, Calcium Channel Blocker; sGC Stimulators, Soluble Guanylate Cyclase Stimulators; COPD, Chronic Obstructive Pulmonary Disease; BNP, B-Type Natriuretic Peptide; MCHC, Mean Corpuscular Hemoglobin Concentration; eGFR, Estimated Glomerular Filtration Rate; LVEF, Left Ventricular Ejection Fraction; AOD, Aortic Diameter; LAD, Left Atrial Diameter; IVSD, Interventricular Septum Diameter; LVEDD, Left Ventricular End Diastolic Diameter; LVPWD, Left Ventricular Posterior Wall Diameter; LVESD, Left Ventricular End Systolic Diameter; BMI, Body Mass Index;*p* < 0.05 indicates a statistically significant difference between the frail and non-frail groups.

To reduce dimensionality and select a parsimonious set of predictors, we employed LASSO logistic regression with 10-fold cross-validation. The optimal regularization parameter (λ) was chosen using the one-standard-error (1-SE) rule, which selects the largest λ within one standard error of the minimum cross-validated error—thereby favoring model simplicity while preserving predictive performance and mitigating overfitting. The selected λ was 0.0238 (S4 Table). At this penalty level, LASSO retained 11 variables with non-zero coefficients: age, serum albumin, eGFR, lymphocyte percentage, MCHC, capacity for action, LVEF, NYHA functional class, history of cerebral infarction, smoking status, and the use of thiazide diuretics. There was no substantial multicollinearity between the variables, with all VIF values<1.5 (S4 Table). These 11 variables constituted the final predictor set for all subsequent modeling. A flowchart illustrating the variable selection process is provided in S1 Fig.

Using the above 11 variables, we developed eight ML models: LR, RF, XGBoost, LightGBM, SVM, CatBoost, NB, and MLP. The hyperparameters of each model were optimized by grid search, and the specific parameters are shown in S5 Table.

### Model performance

**Fig 2:**
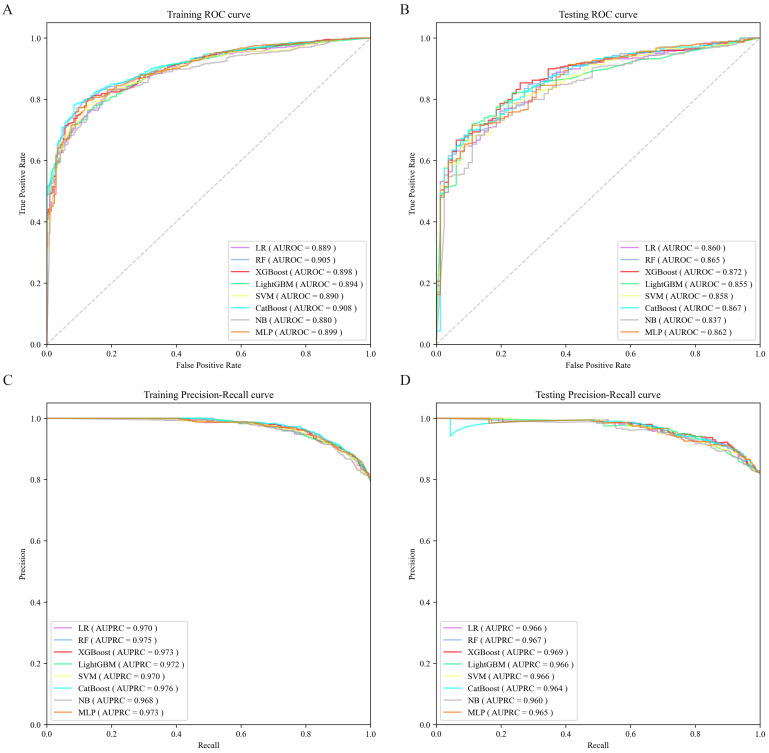
AUROC and AUPRC curves of the eight machine learning models. (A), AUROC curves of the training set; (B), AUROC curves of the testing set; (C), AUPRC curves of the training set; (D), AUPRC curves of the testing set. Abbreviations: LR, Logistic Regression; RF, Random Forest; XGBoost, eXtreme Gradient Boosting; LightGBM, Light Gradient Boosting Machines; SVM, Support Vector Machine; CatBoost, Categorical Boosting; NB, Naive Bayes; MLP, Multilayer Perceptron; AUROC, Area Under The Receiver Operating Characteristic Curve; AUPRC, Area Under Precision-Recall Curve.

**Fig 3:**
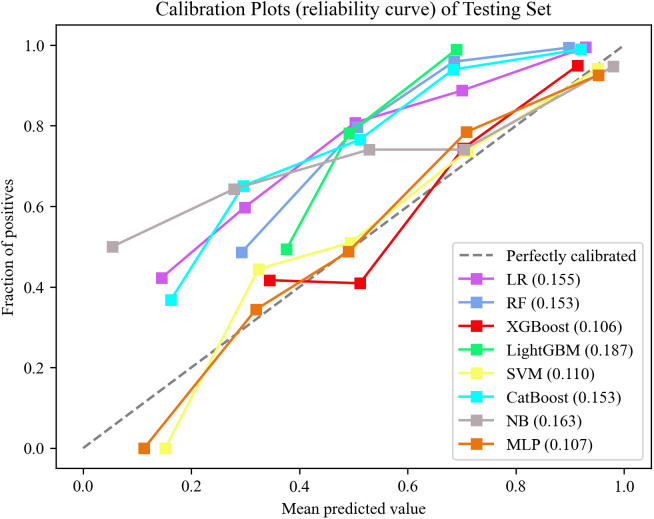
Calibration curves (reliability curves) of eight machine learning models on the testing set. The x-axis represents the mean predicted probability, and the y-axis represents the actual proportion of positive samples. The dashed line represents perfect calibration (where the predicted probability exactly matches the actual proportion). The values in the brackets are the Brier scores of each model, which are used to evaluate the reliability of the model’s probability prediction. Abbreviations: LR, Logistic Regression; RF, Random Forest; XGBoost, eXtreme Gradient Boosting; LightGBM, Light Gradient Boosting Machines; SVM, Support Vector Machine; CatBoost, Categorical Boosting; NB, Naive Bayes; MLP, Multilayer Perceptron.

**Fig 4:**
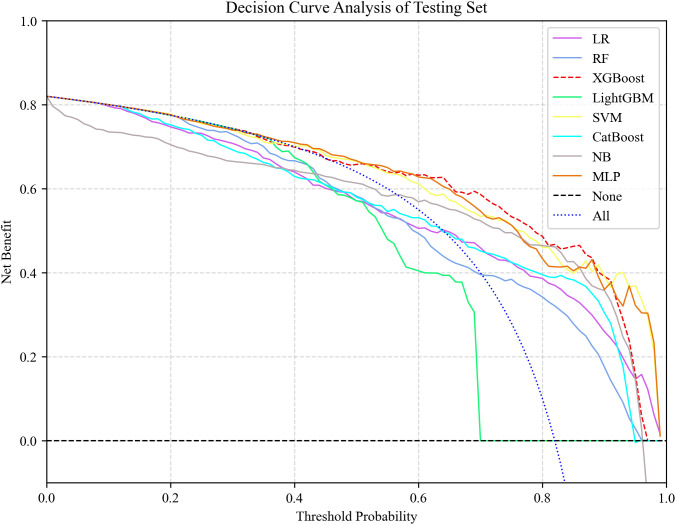
Decision curve analysis of eight machine learning models on the testing set. The x-axis represents the threshold probability, and the y-axis denotes the net benefit. Curves for different models illustrate their net benefit across varying threshold probabilities. The “None” curve (dashed line) assumes no intervention, and the “All” curve (dotted line) assumes all cases are positive. Abbreviations: LR, Logistic Regression; RF, Random Forest; XGBoost, eXtreme Gradient Boosting; LightGBM, Light Gradient Boosting Machines; SVM, Support Vector Machine; CatBoost, Categorical Boosting; NB, Naive Bayes; MLP, Multilayer Perceptron.

The predictive performance of eight machine learning models was evaluated on the testing set. The XGBoost model demonstrated superior overall performance, achieving an AUROC of 0.872 (95% CI: 0.835–0.909) and AUPRC of 0.969 (95% CI: 0.945–0.983) (Fig 2). Calibration performance was evaluated using calibration curves, which assess the agreement between predicted and observed probabilities, together with Brier scores, where lower values indicate better overall probability estimation (Fig 3). The calibration curves indicated that the XGBoost, MLP, and SVM models exhibited relatively better agreement with the ideal 45° reference line than the other models. Among the evaluated models, XGBoost achieved the lowest Brier score (0.106), followed by MLP (0.107) and SVM (0.110), suggesting relatively better overall probability estimation and satisfactory calibration performance than the other evaluated models. DCA revealed that XGBoost provided consistently higher standardized net benefit than other models across the clinically relevant threshold probability range of 60–90% (Fig 4). At the optimal threshold determined by the Youden index, XGBoost maintained strong performance metrics: accuracy (0.787), sensitivity (0.783), specificity (0.802), precision (0.948), recall (0.783), and F1-score (0.858) (Table 2). The training set performance metrics are shown in S6 Table. To further assess the robustness of the XGBoost model, we performed 5-fold stratified cross-validation (random_state = 42) on the full cohort (N = 1,498) using the same set of 11 predictors and hyperparameter configuration selected during the initial development phase. For each performance metric, we report the mean value across the five folds along with a 95% confidence interval estimated via non-parametric bootstrapping with 2,000 resamples (np.random.seed(42)) (S7 Table) The cross-validated performance remained highly consistent with the independent test set results: mean AUROC of 0.873 (vs. 0.872 on testing set), mean AUPRC of 0.967 (vs. 0.969), sensitivity of 0.751 (vs. 0.783), specificity of 0.881 (vs. 0.802), PPV of 0.963 (vs. 0.948), NPV of 0.474 (vs. 0.448), and F1-score of 0.842 (vs. 0.858). The close agreement between these results confirms the robustness of the model against data sampling variability. Based on these comprehensive evaluations, XGBoost was selected as the final prediction model.

**Table 2:** The performance metrics of the eight machine learning models on the testing set.


Model	AUROC(95% CI)	AUPRC(95% CI)	Sensitivity(95% CI)	Specifity(95% CI)	PPV(95% CI)	NPV(95% CI)	F1(95% CI)
LR	0.860 (0.820–0.897)	0.966 (0.951–0.978)	0.824 (0.785–0.862)	0.691 (0.589–0.790)	0.924 (0.894–0.951)	0.463 (0.375–0.549)	0.871 (0.844–0.896)
RF	0.865(0.825–0.902)	0.967(0.951–0.979)	0.775(0.735–0.816)	0.753(0.655–0.841)	0.935 (0.907–0.960	0.424 (0.342–0.500)	0.847 (0.818–0.875)
**XGBoost**	**0.872(0.835–0.907)**	**0.969(0.955–0.980)**	**0.783(0.740–0.824)**	**0.802 (0.711–0.883)**	**0.948 (0.921–0.971**	**0.448 (0.368–0.527)**	**0.858 (0.828–0.884)**
LightGBM	0.855(0.817–0.892)	0.966 (0.947–0.976)	0.734(0.690–0.779)	0.852(0.772–0.928)	0.958 (0.934–0.979)	0.413 (0.341–0.485)	0.831(0.799–0.861)
SVM	0.858 (0.818–0.894)	0.966(0.952–0.978)	0.762(0.719–0.802	0.778(0.681–0.861)	0.940 (0.911–0.964)	0.417 (0.340–0.490)	0.841 (0.812–0.869)
CatBoost	0.867(0.827–0.904)	0.964(0.944–0.980)	0.764(0.722–0.805)	0.778 (0.684–0.863)	0.943 (0.911–0.964)	0.424 (0.343–0.496)	0.843 (0.814–0.870)
NB	0.837(0.793–0.878)	0.960(0.937–0.973)	0.745(0.702–0.787)	0.778(0.686–0.860)	0.939 (0.909–0.963)	0.401 (0.327–0.473)	0.831 (0.800–0.860)
MLP	0.862(0.822–0.898)	0.965 (0.950–0.978)	0.767(0.724–0.808)	0.716 (0.615–0.810)	0.925 (0.894–0.952)	0.403 (0.324–0.479)	0.839 (0.809–0.867)

Abbreviations: LR, Logistic Regression; RF, Random Forest; XGBoost, eXtreme Gradient Boosting; LightGBM, Light Gradient Boosting Machines; SVM, Support Vector Machine; CatBoost, Categorical Boosting; NB, Naive Bayes; MLP, Multilayer Perceptron；AUROC, Area Under The Receiver Operating Characteristic Curve; AUPRC, Area Under Precision-Recall Curve；PPV, positive predictive value; NPV, negative predictive value.

### Model interpretation and application

**Fig 5:**
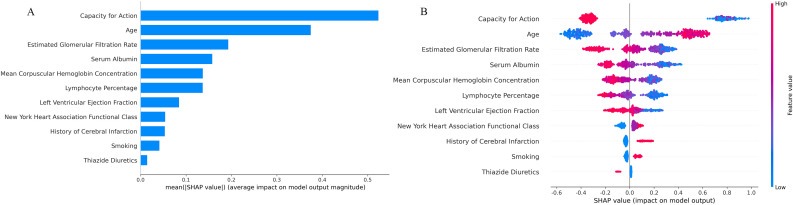
SHAP summary plot for the eleven influential variables in the XGBoost model. (A) The average absolute influence of each factor on the model output magnitude was presented in descending order of feature significance; (B) The graph depicted the dot estimate of the XGBoost model output, with each dot corresponding to a patient in the dataset.

Capacity for action, age, and eGFR were the three most significant factors influencing the prediction of frailty risk, according to an interpretability analysis of the optimal XGBoost model using the SHAP method. These were followed by serum albumin, MCHC, lymphocyte percentage, LVEF, NYHA functional class, history of cerebral infarction, smoking, and the use of thiazide diuretics (Fig 5A). Dependency plot revealed that while capacity for action, eGFR, serum albumin, MCHC, lymphocyte percentage, LVEF, and thiazide diuretics use were negatively associated with the risk of frailty, advanced age, NYHA functional class III-IV, history of cerebral infarction, and smoking were positively associated with the risk of frailty (Fig 5B). We developed an interactive prediction tool (https://frailty-risk-assessment.streamlit.app/) that calculates the probability of frailty risk in patients with HF co-infections in real time through a visual interface and dynamically displays the contributing weights of each clinical variable to provide an interpretable demonstration of model predictions (S2 Fig).

## Discussion

This study developed a machine learning prediction model for the occurrence of frailty in HF patients with acute infections. The prevalence of frailty in our cohort was 80.3%, which is largely consistent with the findings reported by Vidán et al [30]. Notably, this high frailty prevalence leads to class imbalance in the dataset, a factor that may exert non-negligible impacts on model performance and result interpretation. Specifically, a skewed class distribution tends to induce model bias toward the majority class (frail patients), which may inflate the overall accuracy while masking the model’s potential deficiency in identifying the minority class (non-frail patients). Such bias could further lead to misleading judgments about the model’s actual discriminative ability, especially in distinguishing non-frail individuals who may require different clinical management strategies. To mitigate the potential influence of prevalence imbalance on model evaluation, we adopted a comprehensive assessment strategy incorporating discrimination, classification performance, calibration, and clinical utility rather than relying on a single performance metric. The study employed eight machine learning models, with the XGBoost model demonstrating optimal performance. Through XGBoost model analysis, significant predictors associated with frailty were identified, including thiazide diuretics use, serum albumin, eGFR, lymphocyte percentage, MCHC, capacity for action, age, LVEF, NYHA functional class, history of cerebral infarction, and smoking.

This study demonstrated the model’s innovation and practical value through the following aspects: first, the introduced XGBoost model exhibited exceptional predictive performance, with AUROC (0.872) and AUPRC (0.969) metrics significantly outperforming other comparative models. As one of the most widely utilized machine learning models in medical research, XGBoost became a vital tool in clinical decision support systems due to its computational efficiency and superior predictive accuracy, demonstrating significant clinical applicability [31]. Furthermore, the acceptable calibration performance of the XGBoost model supports the potential utility of its predicted probabilities for individualized risk assessment. However, these probability estimates should be further validated in external populations before being applied in routine clinical decision-making. Additionally, this study employed SHAP to enhance the interpretability of the XGBoost model. SHAP analysis provided both global and individual-level explanations by quantifying feature importance, illustrating the directionality of predictor effects, and visualizing patient-specific contributions through waterfall plots integrated into the online risk calculator. This provided reliable evidence-based support for developing personalized treatment strategies. Finally, we successfully developed an interactive web-based application to facilitate real-time clinical data input and visual risk prediction output. This tool significantly improved clinical workflow efficiency and allows clinicians to dynamically monitor disease progression trends, offering immediate decision-making support for precision medicine. In summary, this study extended the predictive model into an interactive visualization tool by integrating a full-process framework of “patient feature input, SHAP-based real-time computation, and visualized output.” This study not only provided a proof-of-concept demonstration of the potential application of machine learning models in clinical risk assessment but also provided a scalable technical approach for advancing intelligent clinical decision-making. Future development of intelligent clinical decision-support tools. However, this tool should be considered a proof-of-concept prototype rather than a clinically ready application, and further external validation is required before clinical implementation.

Compared with previous frailty prediction models for HF and elderly populations, the present study exhibits marked differences in study population, modeling strategy, and predictor composition. Previous models have been predominantly based on logistic regression or nomograms [22,32]. In contrast, our study is the first to focus on patients with HF and acute infections, systematically comparing multiple machine learning algorithms to select the optimal model and incorporating SHAP to enhance interpretability. The core predictors in our study mainly involve medication-, nutrition-, and inflammation-related indicators, which are more innovative than those in prior research. Overall, our model better aligns with the actual clinical needs of this specific patient population. Moreover, the predictors identified in this study implicate both core biological pathways of frailty and disease‑specific predictive factors. Specifically, albumin and lymphocyte percentage reflect nutritional–immune status, renal function and age represent cumulative physiological reserve, and capacity for action integrates multisystem aging effects. Through the interplay of systemic inflammation, nutritional depletion, and hemodynamic stress, these variables may enhance predictive performance in older patients with HF, consistent with previously proposed multidimensional frailty frameworks [33]. In addition, the catabolic syndrome posited by prior studies provides pathophysiological support for the predictive direction of metabolism-related factors observed in our analysis [34].

Currently, there is no universally established gold standard for the frailty prediction probability threshold that triggers intervention. The determination of such a threshold is inherently determined by the specific “risk-benefit ratio” of the intervention itself. Based on the DCA results, the model demonstrated potential clinical utility within a threshold probability range of 0.6 to 0.9 in the present cohort. However, these thresholds should not be interpreted as definitive intervention criteria, as clinical decisions require consideration of individual patient characteristics, intervention risks, and potential benefits. Therefore, practical intervention decisions should integrate multiple factors, including patient preference, overall health status, intervention costs, and associated risks, requiring clinicians to conduct a comprehensive evaluation and balance these considerations. The most feasible strategy is to incorporate the prediction model as an “intelligent early-warning tool” into the clinical workflow, with the final individualized decision made by the physician based on a holistic assessment of the patient’s information. If, upon clinical assessment, a patient is deemed to be at high risk and requires intervention initiation, the following integrated workflow can be implemented: First, the attending physician formulates a personalized intervention plan based on the patient’s specific characteristics. Subsequently, a geriatrician conducts a professional, comprehensive frailty intervention, encompassing nutritional support, exercise guidance, and other components. Finally, the patient’s frailty status is regularly assessed through follow-up to evaluate improvement, and the intervention strategy is dynamically adjusted based on the follow-up findings. The successful implementation of this workflow relies on corresponding resource support: Both the attending physician and the geriatrician must possess substantial clinical experience and appropriate qualifications, while the hospital needs to integrate resources across multiple departments to provide patients with systematic and continuous intervention support.

The findings of this study suggest thiazide diuretics are independently associated with a lower probability. This observation may be attributed to the following potential reasons. This observed association may be attributed to several mechanisms. First, thiazide diuretics primarily reduce cardiac preload and afterload by decreasing blood volume, thereby alleviating symptoms such as dyspnea and edema while improving exercise tolerance [35]. Second, a previous multicenter, randomized, double-blind clinical trial on acute decompensated HF demonstrated that adding oral thiazide diuretics to intravenous loop diuretics mitigated adverse effects like hypokalemia, consequently preventing muscle weakness. This suggests thiazide diuretics may offer more favorable potassium-sparing effects [36]. Furthermore, in infected patients, thiazide diuretics can effectively alleviate fluid exudation caused by primary lesions. Taking pulmonary infection as an example, increased sputum production often leads to persistent or refractory cough, with some patients showing poor response to conventional expectorant therapy. For HF patients during acute infections, thiazide diuretics not only exert their inherent diuretic effects but also significantly improve symptoms of cough and dyspnea associated with pulmonary infection by modulating infection-related fluid exudation mechanisms [37,38]. The explanation above is primarily inferred from the observation that thiazide diuretics can alleviate clinical symptoms in patients with HF and acute infections, which indirectly suggests their use is an independent predictor associated with lower frailty probability. However, this does not provide direct clinical evidence that such drugs can effectively prevent frailty. Therefore, the causal relationship between the two still requires further validation through additional research.

A comparative evaluation provides compelling evidence for the significance of thiazide diuretics. Clinically, loop diuretics, as first-line agents for HF treatment, exhibit significant clinical limitations despite their widespread use. The primary concern is their propensity to induce severe electrolyte imbalances, compounded by the increasingly prevalent phenomenon of diuretic resistance. Research demonstrates that combination therapy not only effectively addresses diuretic resistance but also significantly reduces adverse drug reactions [39]. However, current studies predominantly focus on combining loop diuretics with mineralocorticoid receptor antagonists [40], while the potential of thiazide diuretics remains underinvestigated. Compared to loop diuretics, thiazide diuretics exhibit a more moderate diuretic effect. However, they also carry the risk of causing electrolyte disturbances, which may consequently exacerbate patient frailty. Currently, the combined use of thiazide and loop diuretics has gained acceptance in clinical practice. This dual treatment strategy can synergistically enhance diuretic efficacy, aid in weight reduction, significantly improve edema symptoms, and may potentially reduce the risk of electrolyte imbalances [36]. However, the negative correlation observed in this study between thiazide diuretics and frailty risk is an exploratory finding. It neither supports modifications to current clinical prescribing practices nor carries direct therapeutic implications at this stage. Due to limitations inherent in its cross-sectional design, reliance on single-center data, and the absence of a control group, the current evidence cannot substantiate a preventive effect of the medication on frailty and merely suggests a potential link between the two. Furthermore, in this study, the use of thiazide diuretics was identified based on electronic prescription records, specifically including hydrochlorothiazide and indapamide. Inclusion criteria for medication required prescriptions issued within six months prior to admission, with continuous use documented for six months. However, this study did not record specific dosage ranges or assess patient medication adherence. Future studies should address these limitations through a more detailed investigation.

The findings revealed significantly lower serum albumin levels in the frail group compared to the non-frail group. The relationship between frailty and serum albumin can be explained through the following mechanisms: first, frail elderly individuals exhibit systemic functional decline, including compromised masticatory function. This impairment often leads to inadequate food breakdown during chewing, particularly affecting the digestion and absorption of protein-rich foods. Second, frailty adversely affects gastrointestinal function, further hindering nutrient assimilation. Third, during acute infections, frail patients demonstrate more pronounced protein catabolism compared to their non-frail counterparts [41]. Therefore, for patients with HF complicated by acute infections, nutritional management is critically important. Moreover, micronutrient supplementation (particularly of calcium, iron, zinc, and magnesium) may significantly aid in restoring serum albumin levels [42].

eGFR, a key indicator of renal function, demonstrated an inverse relationship with frailty in this study. A decline in eGFR leads to overactivation of the renin-angiotensin-aldosterone system (RAAS), exacerbating fluid retention. This fluid accumulation is particularly detrimental in HF patients, whose pre-existing fluid overload further restricts mobility and accelerates frailty progression [43]. While eGFR decline is irreversible, the judicious selection of nephroprotective pharmacological agents may help slow the progression of renal insufficiency, thereby delaying the onset of frailty. Furthermore, lymphocyte percentage serves as a key indicator of immune system function, with its decline reflecting impaired immunity and significantly elevating frailty risk [44]. During acute infections, lymphocyte percentage emerges as a clinically significant predictor of frailty among various infection markers. Notably, evidence suggests that enhanced physical activity may prevent immunosenescence and mitigate frailty progression [45].

MCHC, a key diagnostic parameter for anemia, reflects the hemoglobin concentration within red blood cells. A low MCHC often indicates potential iron deficiency in the body. Iron is an essential element for erythropoiesis, and its deficiency can impair hemoglobin synthesis, reducing the oxygen-carrying capacity of red blood cells. This leads to systemic tissue hypoxia, which may manifest as muscle weakness, fatigue, and decreased exercise tolerance [46,47]. Furthermore, chronic hypoxia can disrupt mitochondrial function, decreasing adenosine triphosphate (ATP) production and limiting energy supply for daily activities, thereby accelerating frailty progression [48]. Therefore, increasing dietary iron intake may help improve frailty status in these patients.

This study demonstrates that patients who remain ambulatory are less prone to frailty compared to those who are wheelchair-dependent or bedridden. Therefore, for patients with HF and acute infections, exercise-based rehabilitation should be prioritized to improve mobility. Although physical activity increases myocardial oxygen demand and may transiently worsen HF symptoms, judicious exercise promotes muscle recovery, enhances the capacity for action, and reduces frailty risk—provided the intensity remains within individualized thresholds [49]. This necessitates careful supervision by experienced clinicians to optimize the risk-benefit balance. In summary, personalized exercise prescriptions should be implemented for this population to achieve measurable benefits. In addition, our study found that advanced age significantly increases the likelihood of frailty development, which aligns with previous reports [50]. The study also highlights associations between decreased LVEF, elevated NYHA functional class, history of cerebral infarction, smoking, and elevated frailty risk.

## Limitations

Although this study has yielded significant findings, several limitations should be acknowledged. First, this study cannot establish a causal relationship between the use of thiazide diuretics and the risk of frailty onset. The analysis is further limited by the lack of recorded dosage ranges and assessment of medication adherence. Furthermore, given the retrospective observational design of this study, the observed associations may be subject to multiple confounding factors, including confounding by indication, differences in disease severity, baseline functional status, renal function, physician treatment selection, and concomitant HF therapies. Additionally, due to factors such as acute infectious events and functional decline during hospitalization, the CFS assessment at discharge may be subject to reverse causation. Second, this study did not formally evaluate the inter-rater reliability of the CFS. ADL and IADL assessments primarily rely on self-reported data, which may introduce recall bias, subjective overestimation, or underestimation. Third, the data were derived exclusively from a subset of elderly patients at a specific tertiary class-A public hospital in Nanjing. As a single-center study, the generalizability of the results is inherently limited. Moreover, the predictive model has not been externally validated, and its generalizability across diverse populations requires further confirmation. Therefore, external validation using independent cohorts from multiple institutions is necessary before clinical implementation. Fourth, although missing values were handled using KNN imputation, the missing data mechanism was not formally assessed, and sensitivity analyses using alternative methods were not performed. Future studies should compare different imputation strategies to further validate model robustness. Finally, although class weighting was used to address the high frailty prevalence (80.3%), resampling methods such as SMOTE were not applied to models that lack native class-weighting support, including NB and MLP, which may affect cross-model performance comparisons. Additionally, the model’s generalizability to settings with lower frailty prevalence may be limited.

## Conclusion

This study developed a frailty prediction model for patients with HF complicated by acute infections. The model incorporates 11 readily accessible predictors. This study demonstrated that the use of thiazide diuretics is an independent predictor associated with lower frailty probability. We developed an online calculator as a proof-of-concept tool to demonstrate the potential application of the predictive model and facilitate real-time risk estimation. This tool facilitates the early identification of high-risk individuals and supports the implementation of personalized interventions.

## Supporting information

S1 Fig A flowchart illustrating the variable selection process. NYHA, New York Heart Association; eGFR, estimated glomerular filtration rate; MCHC, mean corpuscular hemoglobin concentration.
(TIF)

S2 Fig The risk web calculator was designed based on the eXtreme Gradient Boosting model. (TIF)

S1 Table The proportion of missing values in variables. (DOCX)

S2 Table Demographics and potential risk factors of patients in the full cohort. (DOCX)

S3 Table Demographics and potential risk factors in the testing and training set. (DOCX)

S4 Table LASSO selection variables and collinearity analysis. (DOCX)

S5 Table The optimal hyperparameters of the eight machine learning models. (DOCX)

S6 Table The performance metrics of the eight machine learning models on the training set. (DOCX)

S7 Table Performance of the XGBoost model on the full cohort based on 5-fold cross-validation. (DOCX)

## Abbreviations

HF: heart failure
CHARLS: China Health and Retirement Longitudinal Study
COPD: chronic obstructive pulmonary disease
NYHA functional class: New York Heart Association functional class
CFS: Clinical Frailty Scale
ADL: Activities of Daily Living
IADL: Instrumental Activities of Daily Living
BMI: body mass index
ARNI: angiotensin receptor neprilysin inhibitor
ACEI: angiotensin-converting enzyme inhibitor
ARB: angiotensin receptor blocker
SGLT-2 inhibitors: sodium-glucose cotransporter protein-2 inhibitors
MRA: mineralocorticoid receptor antagonist
CCB: calcium channel blocker
sGC stimulators: soluble guanylate cyclase stimulators
MCHC: mean corpuscular hemoglobin concentration
eGFR: estimated glomerular filtration rate
BNP: B-type natriuretic peptide
NT-proBNP: N-terminal pro-BNP
LVEF: left ventricular ejection fraction
AOD: aortic diameter
LAD: left atrial diameter
IVSD: interventricular septum diameter
LVEDD: left ventricular end diastolic diameter
LVPWD: left ventricular posterior wall diameter
LVESD: left ventricular end systolic diameter
KNN: K-nearest neighbors
LASSO: least absolute shrinkage and selection operator
VIF: variance inflation factor
AUROC: area under the receiver operating characteristic curve
AUPRC: area under the precision-recall curve
DCA: decision curve analysis
SHAP: SHapley Additive exPlanations
LR: logistic regression
RF: random forest
XGBoost: eXtreme Gradient Boosting
LightGBM: light gradient boosting machine
SVM: support vector machine
CatBoost: categorical boosting
NB: naive bayes
MLP: multilayer perceptron
RAAS: renin-angiotensin-aldosterone system
ATP: adenosine triphosphate
