# Supplementary material for: Frailty prediction in heart failure patients with acute infections: the potential role of thiazide diuretics?
Source: PLoS One. 2026 Aug 12;21(8):e0355848. doi: 10.1371/journal.pone.0355848 (PMC13465814; doi:10.1371/journal.pone.0355848)
Supplement: Supplementary file 1 [file pone.0355848.s003.docx]

| **S1 Table.** The proportion of missing values in variables. | | |
| --- | --- | --- |
| **Variables** | **Missing cases** | **Missing proportion** |
| Body Mass Index | 267 | 17.8% |
| D-Dimer | 90 | 6.0% |
| Estimated Glomerular Filtration Rate | 28 | 1.8% |
| Interleukin-6 | 33 | 2.2% |
| Procalcitonin | 55 | 3.6% |
| C-Reactive Protein | 66 | 4.4% |
