# Supplementary material for: Frailty prediction in heart failure patients with acute infections: the potential role of thiazide diuretics?
Source: PLoS One. 2026 Aug 12;21(8):e0355848. doi: 10.1371/journal.pone.0355848 (PMC13465814; doi:10.1371/journal.pone.0355848)
Supplement: Supplementary file 2 [file pone.0355848.s004.docx]

| **S2 Table.** Demographics and potential risk factors of patients in the full cohort. | | | | |
| --- | --- | --- | --- | --- |
| Variables | Overall (n=1498) | No Frailty(n=295) | Frailty (n=1203) | p |
| Sex, n (%) |  |  |  | 0.033 |
| Female | 602 (40.2) | 102 ( 34.6) | 500 ( 41.6) |  |
| Male | 896 (59.8) | 193 ( 65.4) | 703 ( 58.4) |  |
| Marital Status, n (%) |  |  |  | <0.001 |
| Unmarried | 5 ( 0.3) | 0 ( 0.0) | 5 ( 0.4) |  |
| Divorced And Widowed | 111 ( 7.4) | 3 ( 1.0) | 108 ( 9.0) |  |
| Married | 1382 (92.3) | 292 ( 99.0) | 1090 ( 90.6) |  |
| Literacy, n (%) |  |  |  | 0.061 |
| Illiteracy | 247 (16.5) | 36 ( 12.2) | 211 ( 17.5) |  |
| Primary School | 454 (30.3) | 93 ( 31.5) | 361 ( 30.0) |  |
| Junior High School | 413 (27.6) | 97 ( 32.9) | 316 ( 26.3) |  |
| Senior High School | 239 (16.0) | 41 ( 13.9) | 198 ( 16.5) |  |
| College Degree Or Above | 145 ( 9.7) | 28 ( 9.5) | 117 ( 9.7) |  |
| Capacity For Action, n (%) |  |  |  | <0.001 |
| Bedridden | 469 (31.3) | 4 ( 1.4) | 465 ( 38.7) |  |
| Wheelchair-Dependent | 127 ( 8.5) | 0 ( 0.0) | 127 ( 10.6) |  |
| Ambulatory | 902 (60.2) | 291 ( 98.6) | 611 ( 50.8) |  |
| Smoking, n (%) |  |  |  | 0.001 |
| No | 1008 (67.3) | 224 ( 75.9) | 784 ( 65.2) |  |
| Yes | 490 (32.7) | 71 ( 24.1) | 419 ( 34.8) |  |
| Drinking, n (%) |  |  |  | 0.001 |
| No | 1411 (94.2) | 265 ( 89.8) | 1146 ( 95.3) |  |
| Yes | 87 ( 5.8) | 30 ( 10.2) | 57 ( 4.7) |  |
| NYHA Functional Class, n (%) |  |  |  | <0.001 |
| Ⅱ | 568 (37.9) | 154 ( 52.2) | 414 ( 34.4) |  |
| Ⅲ | 663 (44.3) | 119 ( 40.3) | 544 ( 45.2) |  |
| Ⅳ | 267 (17.8) | 22 ( 7.5) | 245 ( 20.4) |  |
| Medicine, n (%) |  |  |  | 0.133 |
| ＜5 | 600 (40.1) | 130 ( 44.1) | 470 ( 39.1) |  |
| ≥5 | 898 (59.9) | 165 ( 55.9) | 733 ( 60.9) |  |
| ARNI, n (%) |  |  |  | 0.072 |
| No | 1168 (78.0) | 242 ( 82.0) | 926 ( 77.0) |  |
| Yes | 330 (22.0) | 53 ( 18.0) | 277 ( 23.0) |  |
| SGLT-2 Inhibitors, n (%) |  |  |  | 0.153 |
| No | 1213 (81.0) | 248 ( 84.1) | 965 ( 80.2) |  |
| Yes | 285 (19.0) | 47 ( 15.9) | 238 ( 19.8) |  |
| Beta Receptor Blockers, n (%) |  |  |  | 0.551 |
| No | 924 (61.7) | 177 ( 60.0) | 747 ( 62.1) |  |
| Yes | 574 (38.3) | 118 ( 40.0) | 456 ( 37.9) |  |
| MRA, n (%) |  |  |  | 0.057 |
| No | 818 (54.6) | 146 ( 49.5) | 672 ( 55.9) |  |
| Yes | 680 (45.4) | 149 ( 50.5) | 531 ( 44.1) |  |
| ACEI/ARB, n (%) |  |  |  | 0.912 |
| No | 1320 (88.1) | 261 ( 88.5) | 1059 ( 88.0) |  |
| Yes | 178 (11.9) | 34 ( 11.5) | 144 ( 12.0) |  |
| CCB, n (%) |  |  |  | 0.084 |
| No | 1074 (71.7) | 224 ( 75.9) | 850 ( 70.7) |  |
| Yes | 424 (28.3) | 71 ( 24.1) | 353 ( 29.3) |  |
| Loop Diuretics, n (%) |  |  |  | 0.457 |
| No | 888 (59.3) | 181 ( 61.4) | 707 ( 58.8) |  |
| Yes | 610 (40.7) | 114 ( 38.6) | 496 ( 41.2) |  |
| Thiazide Diuretics, n (%) |  |  |  | <0.001 |
| No | 1403 (93.7) | 253 ( 85.8) | 1150 ( 95.6) |  |
| Yes | 95 ( 6.3) | 42 ( 14.2) | 53 ( 4.4) |  |
| Nitrates, n (%) |  |  |  | 0.088 |
| No | 1326 (88.5) | 270 ( 91.5) | 1056 ( 87.8) |  |
| Yes | 172 (11.5) | 25 ( 8.5) | 147 ( 12.2) |  |
| Statins, n (%) |  |  |  | 0.248 |
| No | 775 (51.7) | 162 ( 54.9) | 613 ( 51.0) |  |
| Yes | 723 (48.3) | 133 ( 45.1) | 590 ( 49.0) |  |
| Antiplatelet Drugs, n (%) |  |  |  | 0.351 |
| No | 841 (56.1) | 158 ( 53.6) | 683 ( 56.8) |  |
| Yes | 657 (43.9) | 137 ( 46.4) | 520 ( 43.2) |  |
| Anticoagulants, n (%) |  |  |  | 0.029 |
| No | 1060 (70.8) | 193 ( 65.4) | 867 ( 72.1) |  |
| Yes | 438 (29.2) | 102 ( 34.6) | 336 ( 27.9) |  |
| Cardiac Glycosides, n (%) |  |  |  | 0.013 |
| No | 1402 (93.6) | 286 ( 96.9) | 1116 ( 92.8) |  |
| Yes | 96 ( 6.4) | 9 ( 3.1) | 87 ( 7.2) |  |
| sGC Stimulators, n (%) |  |  |  | 0.396 |
| No | 1484 (99.1) | 294 ( 99.7) | 1190 ( 98.9) |  |
| Yes | 14 ( 0.9) | 1 ( 0.3) | 13 ( 1.1) |  |
| Hypertension, n (%) |  |  |  | 0.92 |
| No | 453 (30.2) | 88 ( 29.8) | 365 ( 30.3) |  |
| Yes | 1045 (69.8) | 207 ( 70.2) | 838 ( 69.7) |  |
| Diabetes Mellitus, n (%) |  |  |  | 0.37 |
| No | 954 (63.7) | 195 ( 66.1) | 759 ( 63.1) |  |
| Yes | 544 (36.3) | 100 ( 33.9) | 444 ( 36.9) |  |
| Hyperlipidemia, n (%) |  |  |  | 0.601 |
| No | 1442 (96.3) | 286 ( 96.9) | 1156 ( 96.1) |  |
| Yes | 56 ( 3.7) | 9 ( 3.1) | 47 ( 3.9) |  |
| Coronary Heart Disease, n (%) |  |  |  | 0.813 |
| No | 689 (46.0) | 138 ( 46.8) | 551 ( 45.8) |  |
| Yes | 809 (54.0) | 157 ( 53.2) | 652 ( 54.2) |  |
| Atrial Fibrillation, n (%) |  |  |  | 0.056 |
| No | 1078 (72.0) | 226 ( 76.6) | 852 ( 70.8) |  |
| Yes | 420 (28.0) | 69 ( 23.4) | 351 ( 29.2) |  |
| Fatty Liver Disease, n (%) |  |  |  | 0.001 |
| No | 1460 (97.5) | 279 ( 94.6) | 1181 ( 98.2) |  |
| Yes | 38 ( 2.5) | 16 ( 5.4) | 22 ( 1.8) |  |
| Cirrhosis, n (%) |  |  |  | 1 |
| No | 1490 (99.5) | 293 ( 99.3) | 1197 ( 99.5) |  |
| Yes | 8 ( 0.5) | 2 ( 0.7) | 6 ( 0.5) |  |
| COPD, n (%) |  |  |  | 0.318 |
| No | 1334 (89.1) | 268 ( 90.8) | 1066 ( 88.6) |  |
| Yes | 164 (10.9) | 27 ( 9.2) | 137 ( 11.4) |  |
| Chronic Cor Pulmonale, n (%) |  |  |  | 0.13 |
| No | 1469 (98.1) | 293 ( 99.3) | 1176 ( 97.8) |  |
| Yes | 29 ( 1.9) | 2 ( 0.7) | 27 ( 2.2) |  |
| Renal Insufficiency, n (%) |  |  |  | <0.001 |
| No | 1092 (72.9) | 259 ( 87.8) | 833 ( 69.2) |  |
| Yes | 406 (27.1) | 36 ( 12.2) | 370 ( 30.8) |  |
| Osteoporosis, n (%) |  |  |  | 0.145 |
| No | 1477 (98.6) | 294 ( 99.7) | 1183 ( 98.3) |  |
| Yes | 21 ( 1.4) | 1 ( 0.3) | 20 ( 1.7) |  |
| Cerebral Infarction, n (%) |  |  |  | <0.001 |
| No | 1137 (75.9) | 259 ( 87.8) | 878 ( 73.0) |  |
| Yes | 361 (24.1) | 36 ( 12.2) | 325 ( 27.0) |  |
| Malignant Tumor, n (%) |  |  |  | 0.558 |
| No | 1387 (92.6) | 276 ( 93.6) | 1111 ( 92.4) |  |
| Yes | 111 ( 7.4) | 19 ( 6.4) | 92 ( 7.6) |  |
| Pulmonary Infection, n (%) |  |  |  | 0.614 |
| No | 80 ( 5.3) | 18 ( 6.1) | 62 ( 5.2) |  |
| Yes | 1418 (94.7) | 277 ( 93.9) | 1141 ( 94.8) |  |
| Urinary Tract Infection, n (%) |  |  |  | 0.094 |
| No | 1427 (95.3) | 287 ( 97.3) | 1140 ( 94.8) |  |
| Yes | 71 ( 4.7) | 8 ( 2.7) | 63 ( 5.2) |  |
| Abdominal Infection, n (%) |  |  |  | 0.803 |
| No | 1462 (97.6) | 289 ( 98.0) | 1173 ( 97.5) |  |
| Yes | 36 ( 2.4) | 6 ( 2.0) | 30 ( 2.5) |  |
| Skin And Soft Tissue Infection, n (%) |  |  |  | 0.095 |
| No | 1474 (98.4) | 294 ( 99.7) | 1180 ( 98.1) |  |
| Yes | 24 ( 1.6) | 1 ( 0.3) | 23 ( 1.9) |  |
| Bloodstream Infection, n (%) |  |  |  | 0.121 |
| No | 1467 (97.9) | 285 ( 96.6) | 1182 ( 98.3) |  |
| Yes | 31 ( 2.1) | 10 ( 3.4) | 21 ( 1.7) |  |
| Sepsis, n (%) |  |  |  | 0.025 |
| No | 1442 (96.3) | 291 ( 98.6) | 1151 ( 95.7) |  |
| Yes | 56 ( 3.7) | 4 ( 1.4) | 52 ( 4.3) |  |
| Septic Shock, n (%) |  |  |  | 0.758 |
| No | 1472 (98.3) | 291 ( 98.6) | 1181 ( 98.2) |  |
| Yes | 26 ( 1.7) | 4 ( 1.4) | 22 ( 1.8) |  |
| BNP, n (%) |  |  |  | <0.001 |
| No | 356 (23.8) | 121 ( 41.0) | 235 ( 19.5) |  |
| Yes | 1142 (76.2) | 174 ( 59.0) | 968 ( 80.5) |  |
| Systolic Blood Pressure, mmHg | 130.00 [119.00, 143.00] | 130.00 [120.00, 140.00] | 130.00 [118.00, 144.50] | 0.394 |
| Diastolic Blood Pressure, mmHg | 76.00 [68.00, 83.00] | 76.00 [70.00, 81.00] | 76.00 [68.00, 84.00] | 0.666 |
| Heart Rate, bpm | 80.00 [72.00, 93.00] | 80.00 [70.00, 90.00] | 82.00 [72.00, 94.00] | 0.005 |
| White Blood Cell, 10^9^/L | 6.84 [5.21, 9.24] | 6.20 [5.05, 8.25] | 7.03 [5.28, 9.42] | <0.001 |
| Lymphocyte Percentage, % | 15.95 [9.40, 23.00] | 21.80 [15.40, 29.05] | 14.30 [8.60, 21.65] | <0.001 |
| Neutrophil Percentage, % | 75.00 [65.50, 83.40] | 68.30 [59.30, 76.40] | 76.60 [67.60, 84.60] | <0.001 |
| Red Blood Cell, 10^9^/L | 3.99 [3.51, 4.45] | 4.25 [3.87, 4.56] | 3.91 [3.45, 4.36] | <0.001 |
| Hemoglobin, g/L | 121.00 [106.00, 134.00] | 129.00 [118.00, 140.00] | 119.00 [103.00, 132.00] | <0.001 |
| MCHC, g/L | 327.00 [319.00, 333.00] | 329.00 [323.00, 335.00] | 326.00 [318.00, 333.00] | <0.001 |
| Platelet, 10^9^/L | 174.00 [131.00, 226.75] | 172.00 [133.50, 227.50] | 174.00 [129.00, 226.00] | 0.631 |
| D-Dimer, ug/mL | 0.90 [0.45, 2.14] | 0.46 [0.25, 0.90] | 1.10 [0.55, 2.46] | <0.001 |
| Alanine Aminotransferase, u/L | 17.25 [12.00, 27.95] | 18.00 [13.00, 26.55] | 17.00 [11.55, 28.00] | 0.112 |
| Aspartate Aminotransferase, u/L | 23.00 [17.00, 32.00] | 21.40 [17.00, 28.00] | 23.40 [17.00, 33.00] | 0.027 |
| Serum Albumin, g/L | 35.20 [31.60, 38.40] | 37.90 [34.90, 39.95] | 34.45 [30.91, 37.80] | <0.001 |
| Total Bilirubin, umol/L | 11.00 [7.70, 16.00] | 10.60 [7.80, 15.45] | 11.10 [7.68, 16.10] | 0.74 |
| Urea, mmol/L, mmol/L | 7.44 [5.59, 10.76] | 6.17 [5.08, 7.96] | 7.86 [5.79, 11.45] | <0.001 |
| Creatinine, umol/L | 85.28 [67.23, 120.65] | 75.80 [63.30, 90.20] | 90.10 [68.85, 127.85] | <0.001 |
| Uric Acid, umol/L | 333.00 [246.00, 442.00] | 317.00 [238.50, 398.82] | 341.36 [248.63, 453.10] | 0.003 |
| Serum Potassium, mmol/L | 3.84 [3.55, 4.17] | 3.80 [3.53, 4.06] | 3.86 [3.55, 4.20] | 0.025 |
| Serum Sodium, mmol/L | 139.50 [136.80, 141.70] | 139.70 [137.90, 141.55] | 139.40 [136.50, 141.80] | 0.081 |
| Total Cholesterol, mmol/L | 3.55 [2.93, 4.32] | 3.81 [3.21, 4.46] | 3.49 [2.88, 4.28] | <0.001 |
| Triglycerides, mmol/L | 1.02 [0.77, 1.42] | 1.10 [0.82, 1.50] | 1.01 [0.76, 1.40] | 0.013 |
| High-Density Lipoprotein, mmol/L | 0.93 [0.76, 1.13] | 0.97 [0.82, 1.13] | 0.92 [0.74, 1.12] | 0.01 |
| Low-Density Lipoprotein, mmol/L | 1.94 [1.45, 2.52] | 2.17 [1.63, 2.61] | 1.88 [1.43, 2.51] | <0.001 |
| eGFR, ml/min | 67.37 [43.70, 84.87] | 84.19 [65.61, 90.52] | 61.94 [40.53, 81.89] | <0.001 |
| Interleukin-6, pg/mL | 16.54 [5.28, 47.33] | 11.12 [3.50, 55.64] | 17.88 [6.02, 45.01] | 0.004 |
| Procalcitonin, pg/mL | 0.10 [0.05, 0.38] | 0.07 [0.04, 0.18] | 0.11 [0.05, 0.42] | <0.001 |
| C-Reactive Protein, mg/L | 18.32 [5.00, 60.34] | 8.14 [2.59, 36.97] | 21.58 [6.27, 66.29] | <0.001 |
| LVEF, % | 61.00 [50.00, 64.00] | 63.00 [57.00, 65.00] | 61.00 [49.00, 63.00] | <0.001 |
| AOD, mm | 34.00 [31.00, 36.00] | 34.00 [31.00, 37.00] | 34.00 [32.00, 36.00] | 0.399 |
| LAD, mm | 44.00 [40.00, 49.00] | 44.00 [39.00, 49.00] | 44.00 [40.00, 49.00] | 0.449 |
| **IVSD**, mm | 10.00 [9.00, 11.00] | 10.00 [9.00, 11.00] | 10.00 [9.00, 11.00] | 0.679 |
| LVEDD, mm | 50.00 [46.00, 55.00] | 50.00 [46.50, 55.50] | 50.00 [46.00, 55.00] | 0.208 |
| LVPWD, mm | 9.00 [9.00, 10.00] | 9.00 [9.00, 10.00] | 9.00 [9.00, 10.00] | 0.325 |
| LVESD, mm | 33.00 [30.00, 40.00] | 33.00 [30.00, 38.00] | 33.00 [30.00, 40.00] | 0.689 |
| Age, year | 77.00 [72.00, 84.00] | 72.00 [68.50, 75.50] | 79.00 [74.00, 85.00] | <0.001 |
| Hospital Days, day | 10.50 [8.00, 15.00] | 11.00 [7.00, 17.00] | 10.00 [8.00, 15.00] | 0.675 |
| BMI, kg/m^2^ | 23.44 [21.07, 25.93] | 24.03 [22.00, 26.04] | 23.29 [20.81, 25.82] | 0.002 |
| **Data are presented as n (%), or median (IQR). Abbreviations: NYHA Functional Class, New York Heart Association Functional Class; ARNI, Angiotensin Receptor Neprilysin Inhibitor; SGLT-2 Inhibitors, Sodium-Glucose Cotransporter Protein-2 Inhibitors; MRA, Mineralocorticoid Receptor Antagonist; ACEI, Angiotensin-Converting Enzyme Inhibitor; ARB, Angiotensin Receptor Blocker; CCB, Calcium Channel Blocker; sGC Stimulators, Soluble Guanylate Cyclase Stimulators; COPD, Chronic Obstructive Pulmonary Disease; BNP, B-Type Natriuretic Peptide; MCHC, Mean Corpuscular Hemoglobin Concentration; eGFR, Estimated Glomerular Filtration Rate; LVEF, Left Ventricular Ejection Fraction; AOD, Aortic Diameter; LAD, Left Atrial Diameter; IVSD, Interventricular Septum Diameter; LVEDD, Left Ventricular End Diastolic Diameter; LVPWD, Left Ventricular Posterior Wall Diameter; LVESD, Left Ventricular End Systolic Diameter; BMI, Body Mass Index.** | | | | |
