# Supplementary material for: Frailty prediction in heart failure patients with acute infections: the potential role of thiazide diuretics?
Source: PLoS One. 2026 Aug 12;21(8):e0355848. doi: 10.1371/journal.pone.0355848 (PMC13465814; doi:10.1371/journal.pone.0355848)
Supplement: Supplementary file 3 [file pone.0355848.s005.docx]

| **S3 Table.** Demographics and potential risk factors in the testing and training set. | | | | |
| --- | --- | --- | --- | --- |
| **Variables** | **Overall (n=1498)** | **Testing set (n=450)** | **Training set (n=1048)** | **p** |
| Sex, n (%) |  |  |  | 0.701 |
| Female | 602 (40.2) | 177 (39.3) | 425 (40.6) |  |
| Male | 896 (59.8) | 273 (60.7) | 623 (59.4) |  |
| Marital Status, n (%) |  |  |  | 0.854 |
| Unmarried | 5 (0.3) | 2 (0.4) | 3 (0.3) |  |
| Divorced And Widowed | 111 (7.4) | 32 (7.1) | 79 (7.5) |  |
| Married | 1382 (92.3) | 416 (92.4) | 966 (92.2) |  |
| Literacy, n (%) |  |  |  | 0.676 |
| Illiteracy | 247 (16.5) | 75 (16.7) | 172 (16.4) |  |
| Primary School | 454 (30.3) | 125 (27.8) | 329 (31.4) |  |
| Junior High School | 413 (27.6) | 129 (28.7) | 284 (27.1) |  |
| Senior High School | 239 (16.0) | 73 (16.2) | 166 (15.8) |  |
| College Degree Or Above | 145 (9.7) | 48 (10.7) | 97 (9.3) |  |
| Capacity For Action, n (%) |  |  |  | 0.787 |
| Bedridden | 469 (31.3) | 146 (32.4) | 323 (30.8) |  |
| Wheelchair-Dependent | 127 (8.5) | 39 (8.7) | 88 (8.4) |  |
| Ambulatory | 902 (60.2) | 265 (58.9) | 637 (60.8) |  |
| Smoking, n (%) |  |  |  | 0.01 |
| No | 1008 (67.3) | 281 (62.4) | 727 (69.4) |  |
| Yes | 490 (32.7) | 169 (37.6) | 321 (30.6) |  |
| Drinking, n (%) |  |  |  | 0.066 |
| No | 1411 (94.2) | 432 (96.0) | 979 (93.4) |  |
| Yes | 87 (5.8) | 18 (4.0) | 69 (6.6) |  |
| NYHA Functional Class, n (%) |  |  |  | 0.119 |
| Ⅱ | 568 (37.9) | 156 (34.7) | 412 (39.3) |  |
| Ⅲ | 663 (44.3) | 217 (48.2) | 446 (42.6) |  |
| Ⅳ | 267 (17.8) | 77 (17.1) | 190 (18.1) |  |
| Medicine, n (%) |  |  |  | 0.753 |
| No | 600 (40.1) | 177 (39.3) | 423 (40.4) |  |
| Yes | 898 (59.9) | 273 (60.7) | 625 (59.6) |  |
| ARNI, n (%) |  |  |  | 1 |
| No | 1168 (78.0) | 351 (78.0) | 817 (78.0) |  |
| Yes | 330 (22.0) | 99 (22.0) | 231 (22.0) |  |
| ACEI/ARB, n (%) |  |  |  | 1 |
| No | 1320 (88.1) | 397 (88.2) | 923 (88.1) |  |
| Yes | 178 (11.9) | 53 (11.8) | 125 (11.9) |  |
| SGLT-2 Inhibitors, n (%) |  |  |  | 0.146 |
| No | 1213 (81.0) | 375 (83.3) | 838 (80.0) |  |
| Yes | 285 (19.0) | 75 (16.7) | 210 (20.0) |  |
| Beta Receptor Blockers, n (%) |  |  |  | 0.243 |
| No | 924 (61.7) | 267 (59.3) | 657 (62.7) |  |
| Yes | 574 (38.3) | 183 (40.7) | 391 (37.3) |  |
| MRA, n (%) |  |  |  | 0.632 |
| No | 818 (54.6) | 241 (53.6) | 577 (55.1) |  |
| Yes | 680 (45.4) | 209 (46.4) | 471 (44.9) |  |
| CCB, n (%) |  |  |  | 0.372 |
| No | 1074 (71.7) | 315 (70.0) | 759 (72.4) |  |
| Yes | 424 (28.3) | 135 (30.0) | 289 (27.6) |  |
| Loop Diuretics, n (%) |  |  |  | 0.239 |
| No | 888 (59.3) | 256 (56.9) | 632 (60.3) |  |
| Yes | 610 (40.7) | 194 (43.1) | 416 (39.7) |  |
| Thiazide Diuretics, n (%) |  |  |  | 0.063 |
| No | 1403 (93.7) | 430 (95.6) | 973 (92.8) |  |
| Yes | 95 (6.3) | 20 (4.4) | 75 (7.2) |  |
| Nitrates, n (%) |  |  |  | 0.056 |
| No | 109 (10.4) | 109 (10.4) | 109 (10.4) |  |
| Yes | 109 (10.4) | 109 (10.4) | 109 (10.4) |  |
| Statins, n (%) |  |  |  | 1 |
| No | 775 (51.7) | 233 (51.8) | 542 (51.7) |  |
| Yes | 723 (48.3) | 217 (48.2) | 506 (48.3) |  |
| Antiplatelet Drugs, n (%) |  |  |  | 0.808 |
| No | 841 (56.1) | 250 (55.6) | 591 (56.4) |  |
| Yes | 657 (43.9) | 200 (44.4) | 457 (43.6) |  |
| Anticoagulants, n (%) |  |  |  | 0.894 |
| No | 1060 (70.8) | 320 (71.1) | 740 (70.6) |  |
| Yes | 438 (29.2) | 130 (28.9) | 308 (29.4) |  |
| Cardiac Glycosides, n (%) |  |  |  | 0.758 |
| No | 1402 (93.6) | 423 (94.0) | 979 (93.4) |  |
| Yes | 96 (6.4) | 27 (6.0) | 69 (6.6) |  |
| sGC Stimulators, n (%) |  |  |  | 1 |
| No | 1484 (99.1) | 446 (99.1) | 1038 (99.0) |  |
| Yes | 14 (0.9) | 4 (0.9) | 10 (1.0) |  |
| Hypertension, n (%) |  |  |  | 0.24 |
| No | 453 (30.2) | 126 (28.0) | 327 (31.2) |  |
| Yes | 1045 (69.8) | 324 (72.0) | 721 (68.8) |  |
| Diabetes Mellitus, n (%) |  |  |  | 0.564 |
| No | 954 (63.7) | 292 (64.9) | 662 (63.2) |  |
| Yes | 544 (36.3) | 158 (35.1) | 386 (36.8) |  |
| Hyperlipidemia, n (%) |  |  |  | 0.324 |
| No | 1442 (96.3) | 437 (97.1) | 1005 (95.9) |  |
| Yes | 56 (3.7) | 13 (2.9) | 43 (4.1) |  |
| Coronary Heart Disease, n (%) |  |  |  | 0.158 |
| No | 689 (46.0) | 194 (43.1) | 495 (47.2) |  |
| Yes | 809 (54.0) | 256 (56.9) | 553 (52.8) |  |
| Atrial Fibrillation, n (%) |  |  |  | 0.738 |
| No | 1078 (72.0) | 327 (72.7) | 751 (71.7) |  |
| Yes | 420 (28.0) | 123 (27.3) | 297 (28.3) |  |
| Fatty Liver Disease, n (%) |  |  |  | 0.976 |
| No | 1460 (97.5) | 438 (97.3) | 1022 (97.5) |  |
| Yes | 38 (2.5) | 12 (2.7) | 26 (2.5) |  |
| Cirrhosis, n (%) |  |  |  | 1 |
| No | 1490 (99.5) | 448 (99.6) | 1042 (99.4) |  |
| Yes | 8 (0.5) | 2 (0.4) | 6 (0.6) |  |
| COPD, n (%) |  |  |  | 0.497 |
| No | 1334 (89.1) | 405 (90.0) | 929 (88.6) |  |
| Yes | 164 (10.9) | 45 (10.0) | 119 (11.4) |  |
| Chronic Cor Pulmonale, n (%) |  |  |  | 0.366 |
| No | 1469 (98.1) | 444 (98.7) | 1025 (97.8) |  |
| Yes | 29 (1.9) | 6 (1.3) | 23 (2.2) |  |
| Renal Insufficiency, n (%) |  |  |  | 0.143 |
| No | 1092 (72.9) | 316 (70.2) | 776 (74.0) |  |
| Yes | 406 (27.1) | 134 (29.8) | 272 (26.0) |  |
| Osteoporosis, n (%) |  |  |  | 0.293 |
| No | 1477 (98.6) | 441 (98.0) | 1036 (98.9) |  |
| Yes | 21 (1.4) | 9 (2.0) | 12 (1.1) |  |
| Cerebral Infarction, n (%) |  |  |  | 0.603 |
| No | 1137 (75.9) | 346 (76.9) | 791 (75.5) |  |
| Yes | 361 (24.1) | 104 (23.1) | 257 (24.5) |  |
| Malignant Tumor, n (%) |  |  |  | 1 |
| No | 1387 (92.6) | 417 (92.7) | 970 (92.6) |  |
| Yes | 111 (7.4) | 33 (7.3) | 78 (7.4) |  |
| Pulmonary Infection, n (%) |  |  |  | 0.526 |
| No | 80 (5.3) | 21 (4.7) | 59 (5.6) |  |
| Yes | 1418 (94.7) | 429 (95.3) | 989 (94.4) |  |
| Urinary Tract Infection, n (%) |  |  |  | 0.31 |
| No | 1427 (95.3) | 433 (96.2) | 994 (94.8) |  |
| Yes | 71 (4.7) | 17 (3.8) | 54 (5.2) |  |
| Abdominal Infection, n (%) |  |  |  | 0.223 |
| No | 1462 (97.6) | 443 (98.4) | 1019 (97.2) |  |
| Yes | 36 (2.4) | 7 (1.6) | 29 (2.8) |  |
| Skin and Soft Tissue Infection, n (%) |  |  |  | 0.75 |
| No | 1474 (98.4) | 444 (98.7) | 1030 (98.3) |  |
| Yes | 24 (1.6) | 6 (1.3) | 18 (1.7) |  |
| Bloodstream Infection, n (%) |  |  |  | 0.638 |
| No | 1467 (97.9) | 439 (97.6) | 1028 (98.1) |  |
| Yes | 31 (2.1) | 11 (2.4) | 20 (1.9) |  |
| Sepsis, n (%) |  |  |  | 0.092 |
| No | 1442 (96.3) | 427 (94.9) | 1015 (96.9) |  |
| Yes | 56 (3.7) | 23 (5.1) | 33 (3.1) |  |
| Septic Shock, n (%) |  |  |  | 0.246 |
| No | 1472 (98.3) | 439 (97.6) | 1033 (98.6) |  |
| Yes | 26 (1.7) | 11 (2.4) | 15 (1.4) |  |
| BNP, n (%) |  |  |  | 0.13 |
| No | 356 (23.8) | 95 (21.1) | 261 (24.9) |  |
| Yes | 1142 (76.2) | 355 (78.9) | 787 (75.1) |  |
| Frailty, n (%) |  |  |  | 0.313 |
| No | 295 (19.7) | 81 (18.0) | 214 (20.4) |  |
| Yes | 1203 (80.3) | 369 (82.0) | 834 (79.6) |  |
| Age, year | 77.00 [72.00, 84.00] | 77.00 [72.00, 84.00] | 77.00 [72.00, 84.00] | 0.92 |
| Hospital Days, day | 10.50 [8.00, 15.00] | 10.00 [8.00, 15.00] | 11.00 [8.00, 16.00] | 0.669 |
| BMI, kg/m^2^ | 23.44 [21.07, 25.93] | 23.53 [21.07, 26.04] | 23.44 [21.08, 25.78] | 0.478 |
| Systolic Blood Pressure, mmHg | 130.00 [119.00, 143.00] | 131.00 [119.00, 144.00] | 130.00 [119.00, 143.00] | 0.509 |
| Diastolic Blood Pressure, mmHg | 76.00 [68.00, 83.00] | 76.00 [70.00, 84.00] | 76.00 [68.00, 83.00] | 0.3 |
| Heart Rate, bpm | 80.00 [72.00, 93.00] | 82.00 [72.00, 92.75] | 80.00 [72.00, 93.00] | 0.99 |
| White Blood Cell, 10^9^/L | 6.84 [5.21, 9.24] | 6.74 [5.11, 9.26] | 6.86 [5.27, 9.20] | 0.943 |
| Lymphocyte Percentage, % | 15.95 [9.40, 23.00] | 15.30 [9.20, 22.10] | 16.10 [9.50, 23.30] | 0.123 |
| Neutrophil Percentage, % | 75.00 [65.50, 83.40] | 75.65 [66.82, 83.50] | 74.60 [65.18, 83.30] | 0.136 |
| Red Blood Cell, 10^9^/L | 3.99 [3.51, 4.45] | 3.95 [3.49, 4.33] | 4.01 [3.52, 4.47] | 0.137 |
| Hemoglobin, g/L | 121.00 [106.00, 134.00] | 120.00 [106.00, 133.00] | 121.00 [105.00, 135.00] | 0.282 |
| MCHC, g/L | 327.00 [319.00, 333.00] | 327.00 [319.00, 334.00] | 326.00 [320.00, 333.00] | 0.606 |
| Platelet, 10^9^/L | 174.00 [131.00, 226.75] | 173.50 [132.00, 225.00] | 174.00 [129.00, 228.00] | 0.861 |
| D-Dimer, ug/mL | 0.90 [0.45, 2.14] | 0.98 [0.49, 2.23] | 0.88 [0.42, 2.11] | 0.195 |
| Alanine Aminotransferase, u/L | 17.25 [12.00, 27.95] | 17.75 [12.55, 28.00] | 17.05 [12.00, 27.08] | 0.282 |
| Aspartate Aminotransferase, u/L | 23.00 [17.00, 32.00] | 23.60 [17.00, 31.90] | 22.00 [17.00, 32.00] | 0.359 |
| Serum Albumin, g/L | 35.20 [31.60, 38.40] | 34.80 [31.89, 38.08] | 35.35 [31.50, 38.50] | 0.27 |
| Total Bilirubin, umol/L | 11.00 [7.70, 16.00] | 10.80 [7.90, 16.00] | 11.16 [7.60, 16.00] | 0.982 |
| Urea, mmol/L | 7.44 [5.59, 10.76] | 7.72 [5.85, 10.89] | 7.35 [5.48, 10.73] | 0.134 |
| Creatinine, umol/L | 85.28 [67.23, 120.65] | 87.54 [69.08, 121.01] | 84.45 [66.38, 120.43] | 0.121 |
| Uric Acid, umol/L | 333.00 [246.00, 442.00] | 348.94 [245.53, 454.75] | 330.00 [246.65, 432.50] | 0.226 |
| Serum Potassium, mmol/L | 3.84 [3.55, 4.17] | 3.85 [3.55, 4.14] | 3.84 [3.55, 4.19] | 0.712 |
| Serum Sodium, mmol/L | 139.50 [136.80, 141.70] | 139.40 [136.62, 141.57] | 139.50 [136.90, 141.80] | 0.397 |
| Total Cholesterol, mmol/L | 3.55 [2.93, 4.32] | 3.52 [2.91, 4.30] | 3.56 [2.94, 4.32] | 0.567 |
| Triglycerides, mmol/L | 1.02 [0.77, 1.42] | 1.03 [0.75, 1.41] | 1.02 [0.78, 1.42] | 0.912 |
| High-Density Lipoprotein, mmol/L | 0.93 [0.76, 1.13] | 0.94 [0.75, 1.15] | 0.93 [0.76, 1.12] | 0.89 |
| Low-Density Lipoprotein, mmol/L | 1.94 [1.45, 2.52] | 1.95 [1.47, 2.49] | 1.94 [1.45, 2.54] | 0.612 |
| eGFR, ml/min | 67.37 [43.70, 84.87] | 64.43 [43.92, 83.73] | 68.32 [43.56, 85.26] | 0.196 |
| Interleukin-6, pg/mL | 16.54 [5.28, 47.33] | 18.46 [5.80, 42.97] | 15.02 [5.03, 50.56] | 0.579 |
| Procalcitonin, pg/mL | 0.10 [0.05, 0.38] | 0.11 [0.05, 0.41] | 0.10 [0.05, 0.35] | 0.197 |
| C-Reactive Protein, mg/L | 18.32 [5.00, 60.34] | 20.86 [5.36, 61.68] | 17.81 [4.86, 59.95] | 0.206 |
| LVEF, % | 61.00 [50.00, 64.00] | 61.00 [51.00, 64.00] | 61.00 [49.75, 64.00] | 0.928 |
| AOD, mm | 34.00 [31.00, 36.00] | 34.00 [32.00, 37.00] | 34.00 [31.00, 36.00] | 0.105 |
| LAD, mm | 44.00 [40.00, 49.00] | 44.00 [40.00, 48.00] | 44.00 [40.00, 49.00] | 0.416 |
| IVSD, mm | 10.00 [9.00, 11.00] | 10.00 [9.00, 11.00] | 10.00 [9.00, 11.00] | 0.55 |
| LVEDD, mm | 50.00 [46.00, 55.00] | 50.00 [46.00, 56.00] | 50.00 [46.00, 55.00] | 0.866 |
| LVPWD, mm | 9.00 [9.00, 10.00] | 9.00 [9.00, 10.00] | 9.00 [9.00, 10.00] | 0.153 |
| LVESD, mm | 33.00 [30.00, 40.00] | 33.00 [30.00, 40.00] | 33.00 [30.00, 40.00] | 0.982 |
| **Data are presented as n (%), or median (IQR). Abbreviations: NYHA Functional Class, New York Heart Association Functional Class; ARNI, Angiotensin Receptor Neprilysin Inhibitor; ACEI, Angiotensin-Converting Enzyme Inhibitor; ARB, Angiotensin Receptor Blocker; SGLT-2 Inhibitors, Sodium-Glucose Cotransporter Protein-2 Inhibitors; MRA, Mineralocorticoid Receptor Antagonist; CCB, Calcium Channel Blocker; sGC Stimulators, Soluble Guanylate Cyclase Stimulators; COPD, Chronic Obstructive Pulmonary Disease; BNP, B-Type Natriuretic Peptide;** BMI, Body Mass Index; **MCHC,** Mean Corpuscular Hemoglobin Concentration；eGFR, Estimated Glomerular Filtration Rate； LVEF, Left Ventricular Ejection Fraction; AOD, Aortic Diameter; LAD, Left Atrial Diameter; IVSD, Interventricular Septum Diameter; LVEDD, Left Ventricular End Diastolic Diameter; LVPWD, Left Ventricular Posterior Wall Diameter; LVESD, Left Ventricular End Systolic Diameter. | | | | |
