# Supplementary material for: Frailty prediction in heart failure patients with acute infections: the potential role of thiazide diuretics?
Source: PLoS One. 2026 Aug 12;21(8):e0355848. doi: 10.1371/journal.pone.0355848 (PMC13465814; doi:10.1371/journal.pone.0355848)
Supplement: Supplementary file 4 [file pone.0355848.s006.docx]

| **S4 Table.** LASSO selection variables and collinearity analysis. | | |
| --- | --- | --- |
| **Variables** | **Coefficient of LASSO** | **VIF** |
| Sex | 0 | / |
| Marital Status | 0 | / |
| Capacity For Action | -1.089291691 | 1.27905581725771 |
| Smoking | 0.006107142 | 1.04463748876795 |
| Drinking | 0 | / |
| NYHA Functional Class | 0.064521449 | 1.15457548737527 |
| MRA | 0 | / |
| CCB | 0 | / |
| Thiazide Diuretics | -0.174604587 | 1.06842119376654 |
| Cardiac Glycosides | 0 | / |
| Fatty Liver Disease | 0 | / |
| Renal Insufficiency | 0 | / |
| Cerebral Infarction | 0.217238094 | 1.03314301978056 |
| BNP | 0 | / |
| Age | 0.084869836 | 1.21913769801126 |
| BMI | 0 | / |
| Heart Rate | 0 | / |
| White Blood Cell | 0 | / |
| Lymphocyte Percentage | -0.008965525 | 1.2294841109636 |
| Neutrophil Percentage | 0 | / |
| Red Blood Cell | 0 | / |
| Hemoglobin | 0 | / |
| MCHC | -0.007626577 | 1.05139785274571 |
| D-Dimer | 0 | / |
| Serum Albumin | -0.025086185 | 1.2548677996523 |
| Urea | 0 | / |
| Creatinine | 0 | / |
| Uric Acid | 0 | / |
| Serum Potassium | 0 | / |
| Total Cholesterol | 0 | / |
| Triglycerides | 0 | / |
| High-Density Lipoprotein | 0 | / |
| Low-Density Lipoprotein | 0 | / |
| eGFR | -0.008715936 | 1.23521419736874 |
| Procalcitonin | 0 | / |
| C-Reactive Protein | 0 | / |
| LVEF | -0.005732550 | 1.19773998876446 |
| **Abbreviations:** LASSO, Least Absolute Shrinkage And Selection Operator; VIF, Variance Inflation Factor; NYHA Functional Class, New York Heart Association Functional Class; MRA, Mineralocorticoid Receptor Antagonist; CCB, Calcium Channel Blocker; BNP, B-Type Natriuretic Peptide; BMI, body mass index; MCHC, Mean Corpuscular Hemoglobin Concentration；eGFR, Estimated Glomerular Filtration Rate; LVEF, Left Ventricular Ejection Fraction. | | |
