# Supplementary material for: Frailty prediction in heart failure patients with acute infections: the potential role of thiazide diuretics?
Source: PLoS One. 2026 Aug 12;21(8):e0355848. doi: 10.1371/journal.pone.0355848 (PMC13465814; doi:10.1371/journal.pone.0355848)
Supplement: Supplementary file 5 [file pone.0355848.s007.docx]

| **S5 Table.** The optimal hyperparameters of the eight machine learning models. | | |
| --- | --- | --- |
| **Model** | **Hyperparameter** | **Optimal value** |
| LR | c | 0.1 |
|  | penalty | L1 |
|  | solver | liblinear |
|  | class weight | balanced |
|  | random state | 1 |
| RF | n estimators | 150 |
|  | max depth | 3 |
|  | min samples leaf | 3 |
|  | min samples split | 8 |
|  | max features | sqrt |
|  | class weight | balanced |
|  | random state | 1 |
| XGBoost | colsample bytree | 0.7 |
|  | gamma | 1 |
|  | learning rate | 0.01 |
|  | max depth | 4 |
|  | n estimators | 300 |
|  | reg alpha | 1 |
|  | reg lambda | 2 |
|  | min child weight | 20 |
|  | subsample | 0.7 |
|  | early stopping rounds | 10 |
|  | class weight | balanced |
|  | random state | 1 |
| LightGBM | colsample bytree | 1 |
|  | learning rate | 0.01 |
|  | max depth | 3 |
|  | n estimators | 50 |
|  | reg alpha | 0.1 |
|  | reg lambda | 0 |
|  | min child samples | 30 |
|  | subsample | 0.8 |
|  | class weight | balanced |
|  | random state | 1 |
| SVM | c | 0.1 |
|  | probability | True |
|  | kernel | linear |
|  | gamma | scale |
|  | class weight | balanced |
|  | random state | 1 |
| CatBoost | iterations | 100 |
|  | max depth | 4 |
|  | learning rate | 0.05 |
|  | l2 leaf reg | 7 |
|  | verbose | False |
|  | auto class weights | Balanced |
|  | border count | 64 |
|  | loss function | Logloss |
|  | random strength | 1 |
|  | random state | 1 |
| MLP | hidden layer sizes | (50,30) |
|  | activation | tanh |
|  | solver | adam |
|  | alpha | 0.0001 |
|  | max iter | 500 |
|  | learning rate init | 0.01 |
|  | early_stopping | True |
|  | random state | 1 |
| NB | priors | None |
|  | var smoothing | 1e-9 |
| **Abbreviations:** LR, Logistic Regression; RF, Random Forest; XGBoost, eXtreme Gradient Boosting; LightGBM, Light Gradient Boosting Machines; SVM, Support Vector Machine; CatBoost, Categorical Boosting; MLP, Multilayer Perceptron; NB, Naive Bayes. | | |
