# Supplementary material for: Frailty prediction in heart failure patients with acute infections: the potential role of thiazide diuretics?
Source: PLoS One. 2026 Aug 12;21(8):e0355848. doi: 10.1371/journal.pone.0355848 (PMC13465814; doi:10.1371/journal.pone.0355848)
Supplement: Supplementary file 6 [file pone.0355848.s008.docx]

| **S6 Table.**The performance metrics of the eight machine learning models on the training set. | | | | | | | |
| --- | --- | --- | --- | --- | --- | --- | --- |
| **Model** | **AUROC** | **AUPRC** | **Sensitivity** | **Specifity** | **PPV** | **NPV** | **F1** |
| LR | 0.889 | 0.970 | 0.826 | 0.808 | 0.944 | 0.544 | 0.881 |
| RF | 0.905 | 0.975 | 0.8 | 0.883 | 0.964 | 0.531 | 0.874 |
| **XGBoost** | **0.898** | **0.973** | **0.801** | **0.874** | **0.961** | **0.530** | **0.874** |
| LightGBM | 0.894 | 0.972 | 0.76 | 0.874 | 0.959 | 0.483 | 0.848 |
| SVM | 0.890 | 0.970 | 0.779 | 0.874 | 0.960 | 0.504 | 0.86 |
| CatBoost | 0.908 | 0.976 | 0.782 | 0.916 | 0.973 | 0.519 | 0.867 |
| NB | 0.880 | 0.968 | 0.765 | 0.874 | 0.959 | 0.488 | 0.851 |
| MLP | 0.899 | 0.973 | 0.772 | 0.902 | 0.968 | 0.504 | 0.859 |
| Abbreviations: LR, Logistic Regression; RF, Random Forest; XGBoost, eXtreme Gradient Boosting; LightGBM, Light Gradient Boosting Machines; SVM, Support Vector Machine; CatBoost, Categorical Boosting; NB, Naive Bayes; MLP, Multilayer Perceptron；AUROC, Area Under The Receiver Operating Characteristic Curve; AUPRC, Area Under Precision-Recall Curve；PPV, positive predictive value; NPV, negative predictive value. | | | | | | | |
