# Supplementary material for: Frailty prediction in heart failure patients with acute infections: the potential role of thiazide diuretics?
Source: PLoS One. 2026 Aug 12;21(8):e0355848. doi: 10.1371/journal.pone.0355848 (PMC13465814; doi:10.1371/journal.pone.0355848)
Supplement: Supplementary file 7 [file pone.0355848.s009.docx]

| **S7 Table**. Performance of the XGBoost model on the full cohort based on 5-fold cross-validation | |
| --- | --- |
| **Metrics** | **5-fold cross-validation mean (95% CI)** |
| AUROC | 0.873(0.843, 0.902) |
| AUPRC | 0.967 (0.958, 0.975) |
| Sensitivity | 0.751 (0.700, 0.811) |
| Specificity | 0.881 (0.834, 0.922) |
| PPV | 0.963 (0.953, 0.975) |
| NPV | 0.474 (0.421, 0.535) |
| F1 | 0.842 (0.810, 0.875) |
| **Abbreviations**: XGBoost, eXtreme Gradient Boosting; 95% CI, 95% confidence interval; AUROC, Area Under The Receiver Operating Characteristic Curve; AUPRC, Area Under Precision-Recall Curve；PPV, positive predictive value; NPV, negative predictive value. | |
