# Supplementary material for: Frailty prediction in heart failure patients with acute infections: the potential role of thiazide diuretics?
Source: PLoS One. 2026 Aug 12;21(8):e0355848. doi: 10.1371/journal.pone.0355848 (PMC13465814; doi:10.1371/journal.pone.0355848)
Supplement: Supplementary file 8 [file pone.0355848.s011.docx]

Dear Editors and Reviewers,

Thank you for giving us the opportunity to submit a revised version of our manuscript titled **“Frailty prediction in heart failure patients with acute infections: the potential role of thiazide diuretics?” (Manuscript ID: PONE-D-26-19524).** We sincerely appreciate the time and effort you have dedicated to reviewing our work, as well as your insightful comments and constructive suggestions. We have carefully considered all the feedback and have made substantial revisions to address the concerns raised. The detailed responses to each comment are provided below, along with an explanation of the corresponding changes made in the manuscript. All modifications in the revised manuscript have been highlighted in red for ease of review.

**Response to Academic Editor**

We thank the academic editor for the valuable suggestions and guidance. We have thoroughly reviewed and revised the entire manuscript to ensure full compliance with the publication requirements of *PLOS ONE*. The specific modifications are as follows:

- We confirm that no custom code was generated or used in this study.
- The funding statement has been updated to clearly indicate that “There was no additional external funding received for this study,” with an added note that “The funders had no role in study design, data collection and analysis, decision to publish, or preparation of the manuscript.”
- The ORCID iDs of the corresponding authors, Jianjun Zou and Yuying Shen, have been verified and are 0000‑0003‑0886‑3153 and 0000-0002-9529-7796, respectively.
- All funding‑related information has been removed from the main text.
- The ethics statement has been relocated to the Methods section.
- All supplementary tables have been removed and are now uploaded separately as “Supporting Information” files, each accompanied by an appropriate caption or description in the main text.
- We have verified the references recommended by the reviewers and confirmed that all are suitable for citation.
- We have carefully checked each reference in the bibliography and found no retracted publications.

**Data availability statement**

This study involves sensitive human participant data. According to the explicit stipulation of our Institutional Review Board (IRB/Ethics Committee, Approval No. KY20250120‑KS‑03), public deposition of the raw individual‑level data is strictly prohibited. Moreover, such public sharing would directly contravene the principle of patient confidentiality. In line with the journal’s own guidelines, we note that an exemption is permitted when “public deposition would violate the research ethics committee‑approved protocol.” Our situation therefore falls precisely within the exemption criteria. In the revised Data Availability Statement, we have clearly stated that while the raw data cannot be made publicly accessible due to privacy constraints, we remain fully committed to scientific transparency. We will make de‑identified aggregate data available to qualified researchers upon reasonable request to the corresponding author, under a formal data access agreement to ensure compliance with ethical standards. We kindly request the editor to consider this exemption. We are prepared to provide any additional documentation if needed. (Revisions are as follows: Page 37, lines 621-622: De-identified aggregated data are available upon reasonable request to the corresponding author, under a data access agreement consistent with ethical standards.)

**Response to Reviewer 1’s Comments**

**Major comments**

**Comment 1:** Interpretation of the association between thiazide diuretics and reduced frailty risk

The main novelty of the manuscript is the identification of thiazide diuretic use as an important predictor associated with lower frailty risk. However, the current wording sometimes implies a possible protective effect, which may overstate the findings.

Because this is a retrospective observational study, the association may be influenced by:

• confounding by indication;

• differences in disease severity;

• baseline functional status;

• renal function;

• physician treatment selection;

• concomitant HF therapies.

Patients receiving thiazide diuretics may represent a subgroup with different clinical characteristics compared with untreated patients.

The authors should:

• clearly state that the finding represents an association rather than causation;

• expand the limitations section regarding residual confounding;

• avoid suggesting that thiazide therapy may prevent or reverse frailty without prospective validation.

**(1) Author's response:** Thank you very much for your valuable suggestion. We fully acknowledge that the cross-sectional/retrospective nature of our study precludes any causal inference regarding a potential preventive effect of thiazide diuretics on frailty. Accordingly, we have revised the relevant sections in the manuscript to ensure the language accurately reflects the associative and exploratory nature of our findings, avoiding any implication of causality. (Revisions are as follows: Page 3, lines 47-48: This study identified the use of thiazide diuretics as an independent predictor associated with lower frailty probability; Page 31, lines 486-487: The findings of this study suggest thiazide diuretics are independently associated with a lower probability; Page 31, lines 501-503: The explanation above is primarily inferred from the observation that thiazide diuretics can alleviate clinical symptoms in patients with HF and acute infections, which indirectly suggests their use is an independent predictor associated with lower frailty probability.)

**(2) Author's response:** Thank you for your insightful comments. We fully agree that, given the observational design of this study, there are inherent limitations in controlling for potential confounding factors—such as multiple confounding factors, including confounding by indication, differences in disease severity, baseline functional status, renal function, physician treatment selection, and concomitant HF therapies. Patients receiving thiazide diuretic therapy may represent a subgroup with distinct clinical characteristics compared to those who did not receive treatment. Consequently, the findings should be interpreted with caution as indicating a statistical association between thiazide diuretics and a reduced risk of frailty, without establishing causality or demonstrating a preventive effect. We have fully addressed these points in the "Limitations" section of the manuscript. (Revisions are as follows: Page 34, lines 574-577: Furthermore, given the retrospective observational design of this study, the observed associations may be subject to multiple confounding factors, including confounding by indication, differences in disease severity, baseline functional status, renal function, physician treatment selection, and concomitant HF therapies.)

**Comment 2:** Definition and timing of frailty assessment

Frailty was assessed using the Clinical Frailty Scale (CFS), with frailty defined as CFS ≥5. The manuscript states that assessments were performed after resolution of acute infection and at discharge. This is important but requires additional clarification.

The authors should explain:

• whether CFS reflected pre-admission baseline frailty or acute illness-related functional decline;

• whether the acute infection itself could temporarily worsen functional status;

• whether CFS assessment at discharge may introduce reverse causality, since hospitalization-related decline may influence frailty classification.

A clearer description of the clinical context of CFS evaluation would improve interpretation.

**Author's response:** We greatly appreciate the reviewer's comments and fully concur with them. The frailty assessment in our study was conducted at hospital discharge, and therefore reflects the frailty status at that time. We also agree that the CFS assessment at discharge may be subject to reverse causation, owing to factors such as functional decline during hospitalization. In addition, both the acute infectious event and the hospitalization itself may unavoidably and temporarily worsen the patient's functional status. These two points have now been added to the Limitations section of the manuscript. (Page 34-35, lines 578-580: “Additionally, due to factors such as acute infectious events and functional decline during hospitalization, the CFS assessment at discharge may be subject to reverse causation.”)

**Comment 3:** Machine learning methodology and model validation

The methodological approach is generally appropriate; however, some aspects require clarification.

(1)The authors should provide more details regarding:

• whether feature selection was performed strictly within the training dataset to avoid information leakage;

• whether preprocessing steps (including KNN imputation and scaling) were performed only on the training set before application to the test set;

• whether hyperparameter optimization was nested within cross-validation.

(2)Given that the dataset originates from a single institution, external validation is necessary before clinical implementation.

(3)The authors should emphasize that the online calculator represents a proof-of-concept tool rather than a clinically ready application.

**Author’s response:**

(1)We thank the reviewer for this important comment regarding potential information leakage during machine learning model development.

In the original manuscript, we stated that the dataset was randomly split into training and testing sets in a 7:3 ratio. The training set was used for variable selection and model development, whereas the testing set was reserved for independent evaluation of model performance.(Page 12, lines 244-246: “The dataset was randomly split(set.seed(42)) into training and testing sets in a 7:3 ratio. The training set was used for variable selection and model development, while the testing set evaluated model performance.”)

To further clarify the workflow, we have revised the Methods section to explicitly state that all feature selection procedures, including univariate analysis, LASSO regression, and VIF assessment, were performed exclusively within the training dataset. Similarly, preprocessing procedures, including KNN imputation and Z-score standardization, were conducted using only the training dataset. The imputation strategy and scaling parameters derived from the training set were subsequently applied to the independent testing set.

(Page 11, lines 224-228: “Missing data were imputed using the K-nearest neighbors (KNN) algorithm after splitting the dataset into training and testing sets. The optimal k value (k=5) was determined by minimizing the root mean square error (RMSE) of imputation performance, assessed via internal cross-validation within the training set. The KNN imputation model was fitted exclusively on the training set, and the learned imputation strategy was subsequently applied to the testing set.”)

(Page 12, lines 246-252: “Continuous variables were standardized using Z-score normalization based on the mean and standard deviation calculated from the training dataset, and the same scaling parameters were subsequently applied to the testing dataset.~~c~~Categorical variables were encoded via one-hot encoding. All feature selection procedures were performed exclusively within the training dataset. Variables significantly associated with frailty in univariate analysis (p < 0.05) were retained for subsequent LASSO regression.”)

Hyperparameter optimization was performed using 10-fold cross-validation within the training dataset rather than nested cross-validation. For each candidate hyperparameter configuration, the mean ROC-AUC across the 10 validation folds was calculated, and the configuration with the highest average performance was selected. The independent testing dataset was not involved in hyperparameter optimization or model selection. After determining the optimal hyperparameters, the final model was retrained using the entire training dataset and subsequently evaluated on the independent testing dataset. We have added corresponding descriptions in the revised Methods section to clarify that the independent testing set was not involved in hyperparameter optimization or model selection, and that the final model performance was subsequently evaluated on the independent testing set.

(Page 13, lines 276-281: “For each candidate hyperparameter configuration, we computed the mean ROC-AUC across the 10 validation folds using scikit-learn (v1.2.2) and selected the configuration with the highest average performance. The independent testing set was not involved in hyperparameter optimization or model selection. The final model was then retrained on the full training set using these optimal hyperparameters, with random_state=1 to ensure reproducibility, and its performance was subsequently evaluated on the independent testing set.”)

(2)We sincerely thank the reviewer for highlighting the importance of external validation for clinical implementation. We agree that external validation using independent cohorts from different institutions and populations is essential to evaluate the generalizability and clinical applicability of the developed model.

In the revised manuscript, we have further emphasized this limitation in the Discussion section. Specifically, we have stated that our study was conducted using data from a single institution, and the predictive model has not yet undergone external validation. Therefore, further validation in diverse populations and independent clinical settings is required before potential clinical implementation.

(Page 35, lines 582-587: “Third, the data were derived exclusively from a subset of elderly patients at a specific tertiary class-A public hospital in Nanjing. As a single-center study, the generalizability of the results is inherently limited. Moreover, the predictive model has not been externally validated, and its generalizability across diverse populations requires further confirmation. Therefore, external validation using independent cohorts from multiple institutions is necessary before clinical implementation.”)

(3)We sincerely thank the reviewer for this valuable suggestion. We agree that the online calculator should be interpreted cautiously, particularly given that the model was developed using data from a single institution and has not yet undergone external validation.

Accordingly, we have revised the manuscript to clarify that the online calculator represents a proof-of-concept tool developed to demonstrate the potential application of the predictive model, rather than a clinically ready application for direct decision-making. We have also modified the relevant descriptions to avoid overstating its clinical applicability and emphasized that further external validation is required before clinical implementation.

(Page 3, lines 48-49 and Page 35, lines 599-601: “We developed an online calculator as a proof-of-concept tool to demonstrate the potential application of the predictive model and facilitate real-time risk estimation.”)

(Page 28-29, lines 436-445: “In summary, this study extended the predictive model into an interactive visualization tool by integrating a full-process framework of “patient feature input, SHAP-based real-time computation, and visualized output.” This study provided a proof-of-concept demonstration of the potential application of machine learning models in clinical risk assessment but also provided a scalable technical approach for future development of intelligent clinical decision-support tools. However, this tool should be considered a proof-of-concept prototype rather than a clinically ready application, and further external validation is required before clinical implementation.”)

**Comment 4:** High prevalence of frailty and model calibration

Frailty prevalence was very high (80.3%), which reflects the selected population but may influence model performance metrics. The authors appropriately considered AUROC and AUPRC; however, calibration becomes particularly important in this context.

The manuscript should provide:

• more detailed calibration results;

• discussion of potential impact of prevalence imbalance;

• clarification of whether predicted probabilities are reliable for clinical decision-making.

**Author’s Response:**

We sincerely thank the reviewer for this insightful comment regarding the impact of high frailty prevalence and the importance of calibration assessment. We agree that calibration is particularly important in populations with a high prevalence of the outcome, as class imbalance may affect model performance evaluation and the interpretation of predicted probabilities.

In the revised manuscript, we have expanded the description of calibration performance in the Results section. Specifically, we added a more detailed evaluation based on calibration curves and Brier scores. The revised text describes the agreement between predicted and observed probabilities, the relative performance of different models based on calibration curves, and the Brier scores of each model. The XGBoost model demonstrated relatively better calibration performance, with the lowest Brier score (0.106) among the evaluated models. (Page 21, lines 335-342: “Calibration performance was evaluated using calibration curves, which assess the agreement between predicted and observed probabilities, together with Brier scores, where lower values indicate better overall probability estimation (Fig. 3). The calibration curves indicated that the XGBoost, MLP, and SVM models exhibited relatively better agreement with the ideal 45° reference line than the other models. Among the evaluated models, XGBoost achieved the lowest Brier score (0.106), followed by MLP (0.107) and SVM (0.110), suggesting relatively better overall probability estimation and satisfactory calibration performance than the other evaluated models.”)

In addition, we have further discussed the potential impact of the high prevalence of frailty (80.3%) and resulting class imbalance on model evaluation in the Discussion section. We clarified that an imbalanced outcome distribution may influence model behavior and potentially bias performance assessment. To mitigate the potential influence of prevalence imbalance on model evaluation, we adopted a comprehensive assessment strategy incorporating discrimination, classification performance, calibration, and clinical utility rather than relying on a single performance metric. (Page 27, lines 402-413: “This study developed a machine learning prediction model for the occurrence of frailty in HF patients with acute infections. The prevalence of frailty in our cohort was 80.3%, which is largely consistent with the findings reported by Vidán et al. Notably, this high frailty prevalence leads to class imbalance in the dataset, a factor that may exert non-negligible impacts on model performance and result interpretation. Specifically, a skewed class distribution tends to induce model bias toward the majority class (frail patients), which may inflate the overall accuracy while masking the model's potential deficiency in identifying the minority class (non-frail patients). Such bias could further lead to misleading judgments about the model's actual discriminative ability, especially in distinguishing non-frail individuals who may require different clinical management strategies. To mitigate the potential influence of prevalence imbalance on model evaluation, we adopted a comprehensive assessment strategy incorporating discrimination, classification performance, calibration, and clinical utility rather than relying on a single performance metric.”)

Furthermore, we have clarified the interpretation of predicted probabilities generated by the model. The predicted probabilities should be considered individualized risk estimates rather than definitive clinical decision thresholds. Although the model demonstrated acceptable calibration performance within the present cohort, further external validation in independent populations with different frailty prevalence is required before clinical implementation. The online prediction tool has therefore been described as a proof-of-concept prototype rather than a clinically ready decision-support system.

(Page 30, lines 462-485: Currently, there is no universally established gold standard for the frailty prediction probability threshold that triggers intervention. The determination of such a threshold is inherently determined by the specific "risk-benefit ratio" of the intervention itself. Based on the DCA results, the model demonstrated potential clinical utility within a threshold probability range of 0.6 to 0.9 in the present cohort. However, these thresholds should not be interpreted as definitive intervention criteria, as clinical decisions require consideration of individual patient characteristics, intervention risks, and potential benefits. Therefore, practical intervention decisions should integrate multiple factors, including patient preference, overall health status, intervention costs, and associated risks, requiring clinicians to conduct a comprehensive evaluation and balance these considerations. The most feasible strategy is to incorporate the prediction model as an "intelligent early-warning tool" into the clinical workflow, with the final individualized decision made by the physician based on a holistic assessment of the patient's information. If, upon clinical assessment, a patient is deemed to be at high risk and requires intervention initiation, the following integrated workflow can be implemented: First, the attending physician formulates a personalized intervention plan based on the patient's specific characteristics. Subsequently, a geriatrician conducts a professional, comprehensive frailty intervention, encompassing nutritional support, exercise guidance, and other components. Finally, the patient's frailty status is regularly assessed through follow-up to evaluate improvement, and the intervention strategy is dynamically adjusted based on the follow-up findings. The successful implementation of this workflow relies on corresponding resource support: Both the attending physician and the geriatrician must possess substantial clinical experience and appropriate qualifications, while the hospital needs to integrate resources across multiple departments to provide patients with systematic and continuous intervention support.)

**Minor comments**

**Comment 1:** Abstract

The conclusion in the abstract should be moderated. The sentence:

“This study demonstrated that the use of thiazide diuretics was associated with reduced frailty risk” should be revised to: “This study identified thiazide diuretic use as an independent predictor associated with lower frailty probability.”

This better reflects the observational design.

**Author's response:** We greatly appreciate the reviewer's insightful suggestions and fully agree with their perspective. We concur that the conclusion in the abstract regarding the association between thiazide diuretics and frailty should be expressed more cautiously, and we have accordingly revised the wording in the main text to moderate the statement. (Revisions are as follows: Page 3, lines 47-48: This study identified the use of thiazide diuretics as an independent predictor associated with lower frailty probability.)

**Comment 2:** Introduction

The introduction is comprehensive but could be shortened. Some statements regarding machine learning as a “virtual clinician” are somewhat exaggerated and should be toned down to maintain scientific neutrality.

**Author's response:** We thank the reviewer for the valuable suggestion and agree that the analogy comparing machine learning to a “virtual clinician” in the original manuscript was inappropriate and overstated. Accordingly, we have removed that content from the manuscript. (The deleted content is as follows: Page 6-7, lines 128-132:They can efficiently process vast amounts of clinical data, offering distinct advantages in disease prediction, multivariate analysis, and personalized treatment—effectively serving as an additional reliable “virtual clinician” for patients. This not only enhances clinicians’ confidence but also ensures more comprehensive and professional disease management for patients.)

**Comment 3:** Methods – inclusion criteria

The exclusion of NYHA class I patients should be justified more clearly, as this criterion may introduce selection bias.

**Author's response:** We thank the reviewer for raising this issue. The rationale for excluding NYHA class I patients is as follows: NYHA class I is defined as “no limitation of physical activity; ordinary physical activity does not cause undue fatigue, palpitation, dyspnea, or anginal pain.” Patients in this class have significantly milder disease and higher functional reserve, and they differ systematically from NYHA class II–IV patients in terms of baseline characteristics, medication regimens, and prognosis. Including such patients would potentially increase the difficulty of controlling for confounding by indication. Therefore, we proactively excluded this subgroup to ensure relative homogeneity of the study cohort in terms of disease severity, thereby enhancing the internal validity of our multivariable association analyses.

**Comment 4:** Missing data handling

The authors used KNN imputation for missing values. Please clarify:

• whether missingness was assumed to be missing completely at random (MCAR), missing at random (MAR), or not assessed;

• whether sensitivity analyses using alternative imputation methods were considered.

**Author’s Response:**

We appreciate the reviewer’s insightful comment regarding the mechanism of missing data, which is important for evaluating the validity of the imputation strategy.

We first examined the patterns and proportions of missing values for all variables. As shown in Supplementary Table S1, the proportion of missing values ranged from 1.8% to 17.8%. The highest missing proportion was observed for body mass index (BMI) (17.8%), while other variables, including D-dimer, estimated glomerular filtration rate (eGFR), interleukin-6, procalcitonin, and C-reactive protein, had missing proportions below 6%.

The missingness mechanism was not formally tested in the present study. However, considering the clinical characteristics of patients with heart failure, missing values may be related to observed clinical factors, such as disease severity, patient condition, routine examination procedures, and healthcare practices. These factors were partially captured by the variables included in our dataset. Therefore, based on the observed data characteristics and clinical considerations, we assumed that the missing data mechanism was Missing at Random (MAR), meaning that the probability of missingness depends on observed variables rather than the missing values themselves.

Under this assumption, K-nearest neighbor (KNN) imputation was applied to handle missing values, as this approach can utilize similarities among observed samples and preserve potential relationships among predictors.

We acknowledge that the missingness mechanism was not formally verified and that sensitivity analyses using alternative imputation approaches were not performed. This limitation has been added to the revised manuscript. Future studies should further evaluate different missing data assumptions and compare multiple imputation strategies to confirm the robustness of the prediction model. (Page 35, lines 587-590: “Fourth, although missing values were handled using KNN imputation, the missing data mechanism was not formally assessed, and sensitivity analyses using alternative methods were not performed. Future studies should compare different imputation strategies to further validate model robustness.”)

**Comment 5:** Feature selection

The manuscript combines univariate analysis followed by LASSO selection. This approach may exclude clinically relevant variables that are not statistically significant individually but contribute to multivariable prediction.

The authors should briefly justify this strategy.

**Author’s Response:**

We appreciate the reviewer’s valuable comment regarding the feature selection strategy. We agree that univariate screening may potentially exclude variables that are not statistically significant individually but may contribute to multivariable prediction.

In this study, univariate analysis was used as an initial screening step to reduce the dimensionality of candidate variables and to improve computational efficiency given the relatively large number of clinically relevant variables considered. Subsequently, LASSO logistic regression with 10-fold cross-validation was applied to further select the most informative predictors and reduce the risk of overfitting. The final predictor set was determined based on the non-zero coefficients identified by LASSO rather than univariate analysis alone. We have clarified the rationale for this feature selection strategy in the revised manuscript. (Page 16, lines 313-315: “Variables showing significant associations in univariate analyses (p < 0.05) were retained as candidate predictors for subsequent LASSO-based selection, resulting in 37 candidate predictors. ”)

**Comment 6:** SHAP interpretation

The SHAP analysis is a valuable component of the study. However, the authors should clarify whether SHAP values represent:

• global feature importance;

• directionality of association;

• individual patient-level interpretation.

**Author’s Response:**

We appreciate the reviewer’s valuable comment regarding the interpretation of SHAP values. In this study, SHAP analysis was used for both global and individual-level interpretation of the XGBoost model.

First, the mean absolute SHAP values were used to quantify the relative contribution of each predictor to the model output and identify the most important features at the population level (global feature importance, Fig. 5A). Second, SHAP dependency plots were used to illustrate the directionality of predictor effects, indicating whether specific variables increased or decreased the predicted probability of frailty (Fig. 5B).

Third, individual-level SHAP waterfall plots were incorporated into the online risk calculator to provide patient-specific explanations by showing how each feature contributed to an individual prediction. We have clarified these aspects of SHAP interpretation in the revised manuscript. (Page 28, lines 427-430: “SHAP analysis provided both global and individual-level explanations by quantifying feature importance, illustrating the directionality of predictor effects, and visualizing patient-specific contributions through waterfall plots integrated into the online risk calculator.”)

**Comment 7:**Figures and supplementary material

The figures should be checked to ensure that:

• axes and legends are sufficiently explained;

• abbreviations are defined;

• readers without machine learning expertise can interpret the results.

**Author’s response:**

We thank the reviewer for this helpful suggestion. We carefully reviewed all figures and supplementary materials. The axes and legends are fully labeled, abbreviations are defined in the corresponding figure legends or at their first appearance in the manuscript, and explanatory information has been provided to facilitate interpretation by readers without a machine learning background. We have rechecked all figures and confirmed their consistency and clarity in the revised manuscript.

**Comment 8: Discussion**

**The discussion should include a clearer comparison with previous frailty prediction models in HF and geriatric populations.**

**The authors should also discuss whether the identified predictors (albumin, renal function, lymphocyte percentage, functional capacity, age) reflect known biological pathways of frailty rather than disease-specific predictors.**

**I strongly suggest to see and discuss the following references:**

**Multidimensional frailty: Testa G et al., Multidimensional frailty evaluation in elderly outpatients with chronic heart failure: A prospective study. Eur J Prev Cardiol. 2019 Jul;26(10):1115-1117.**

**Low arterial pressure and catabolic syndrome: Curcio F et al. . The reverse metabolic syndrome in the elderly: Is it a "catabolic" syndrome? Aging Clin Exp Res. 2018 Jun;30(6):547-554.**

**Author's response:** We sincerely thank the reviewer for the valuable suggestions. We agree that the Discussion section indeed needs to include a comparison between our model and previous frailty prediction models in heart failure and elderly populations. Compared with existing studies, our study has clear advantages in terms of study population, modeling strategies, and predictor selection. Moreover, the predictors identified in our study not only encompass known biological pathways of frailty but also reflect disease‑specific predictive factors. To illustrate this point, we have cited the prospective study by Testa et al. (2019) on multidimensional frailty evaluation in elderly outpatients with chronic heart failure, as well as the work by Curcio et al. (2018) on low arterial pressure and catabolic syndrome. These comparisons and discussions have been added to the Discussion section to strengthen the completeness and persuasiveness of our argument. (Revisions are as follows: Page 29, lines 446-461: Compared with previous frailty prediction models for HF and elderly populations, the present study exhibits marked differences in study population, modeling strategy, and predictor composition. Previous models have been predominantly based on logistic regression or nomograms. In contrast, our study is the first to focus on patients with HF and acute infections, systematically comparing multiple machine learning algorithms to select the optimal model and incorporating SHAP to enhance interpretability. The core predictors in our study mainly involve medication-, nutrition-, and inflammation-related indicators, which are more innovative than those in prior research. Overall, our model better aligns with the actual clinical needs of this specific patient population. Moreover, the predictors identified in this study implicate both core biological pathways of frailty and disease‑specific predictive factors. Specifically, albumin and lymphocyte percentage reflect nutritional–immune status, renal function and age represent cumulative physiological reserve, and capacity for action integrates multisystem aging effects. Through the interplay of systemic inflammation, nutritional depletion, and hemodynamic stress, these variables may enhance predictive performance in older patients with HF, consistent with previously proposed multidimensional frailty frameworks. In addition, the catabolic syndrome posited by prior studies provides pathophysiological support for the predictive direction of metabolism-related factors observed in our analysis.)

**Comment 9: Data availability statement**

**The current data availability statement appears inconsistent with PLOS ONE requirements. Since clinical data were analyzed, the authors should clarify whether anonymized data can be made available through an appropriate repository or upon reasonable request, according to ethical restrictions.**

**Author's response:** We thank the reviewer for the reminder. We have revised our Data Availability Statement accordingly. However, due to ethical restrictions, the raw individual‑level data cannot be made publicly available. De‑identified aggregated data are accessible to qualified researchers upon reasonable request. (Revisions are as follows: Page 37, lines 621-622: De-identified aggregated data are available upon reasonable request to the corresponding author, under a data access agreement consistent with ethical standards.)

**Response to Reviewer 2’s Comments**

**Comment 1:** reducing redundancy by approximately 20–30%, improving transitions between paragraphs, shortening overly long explanatory sections, consolidating repeated descriptions of previous studies' limitations, and making the rationale progress more directly from the clinical problem to the need for an interpretable prediction model. I estimate these revisions could reduce the manuscript by 1,000–1,500 words while making it more concise and easier to follow, without sacrificing scientific rigor.

ie Some background paragraphs explain concepts already familiar to most readers.

**Author's response:** We sincerely thank the reviewer for the suggestions. We agree that the original manuscript contained excessive redundancy. We have improved the transitions between paragraphs, shortened overlong explanatory paragraphs, and consolidated repetitive descriptions of previous study limitations, so that the progression from clinical questions to the need for an interpretable prediction model is now more direct. In addition, we have deleted and optimized redundant content in the Introduction and Discussion sections. The optimized content is as follows: Page 4, lines 65-72: (1) However, previous research on frailty has primarily focused on stable chronic disease populations [7]. However, frailty-related risk factors in patients with coexisting acute and chronic conditions—a common yet understudied clinical scenario—remain largely unexplored. In HF with acute infection, the overlapping pathophysiology of fluid retention (HF) and fluid exudation (infection) may synergistically exacerbate fluid overload, manifesting as bilateral lower extremity edema and secondary muscle strength decline, thereby increasing the risk of prolonged bed rest and adverse outcomes[8]. These complications not only worsen prognosis but also impose substantial challenges to clinical management[9, 10].

(2) Page 5, lines 92-103: Moreover, existing frailty prediction models, whether based on conventional regression or machine learning approaches, have notable limitations. Logistic regression-based nomograms, while achieving acceptable discrimination (e.g., AUCs of 0.912 and 0.881 in diabetic patients[14]; AUC of 0.847 in elderly coronary heart disease patients[15]), often suffer from limited practical utility or small sample sizes. Machine learning-based models, such as an XGBoost model for chronic obstructive pulmonary disease (COPD) patients using China Health and Retirement Longitudinal Study (CHARLS) data (test AUC 0.942)[16], have demonstrated superior performance but frequently lack objective laboratory parameters. Moreover, most existing models overlook model interpretability—a critical barrier to clinical adoption, as clinicians require intuitive understanding of how individual predictors contribute to frailty risk at the patient level. SHAP (SHapley Additive exPlanations), a game theory-based approach, quantifies each feature's marginal contribution to individual predictions, effectively bridging the gap between predictive accuracy and clinical transparency[17, 18].

The following content has been deleted from the Introduction section (Page 4, lines 61-64): Additionally, clinical observations indicate that hospitalized HF patients frequently present with concurrent acute infections. This comorbid condition may lead to prolonged hospital stays, increased short-term readmission rates, and elevated post-discharge mortality.

**Comment 2:** For example, detailed descriptions of machine learning visualization tumor imaging examples are not essential.

The paragraph discussing tumor imaging can probably be removed entirely because it does not directly support the rationale of this study. Instead, simply state that SHAP improves model interpretability.

**Author's response:** We greatly appreciate the reviewer’s valuable suggestions. The detailed descriptions of machine learning visualizations of tumor imaging examples are not essential; we have therefore removed them and replaced them with a brief statement on the role of SHAP in enhancing model interpretability. (The deleted content is as follows: Page 7, lines 132-134: For instance, in tumor imaging analysis, machine learning visualization techniques can precisely identify malignant lesion areas, with interpretability that aligns with pathological findings, thereby significantly improving clinical trust.)

Additional Revisions:

Beyond the specific comments above, we have also undertaken a thorough proofreading of the manuscript to improve language clarity and consistency. All figure captions have been double-checked for accuracy, and any minor typographical errors have been corrected.

Once again, we thank you and the reviewers for your thoughtful feedback, which has significantly improved the quality of our work. We hope that the revised manuscript now meets the journal’s standards for publication.

Sincerely,

On behalf of all authors
