# Supplementary material for: Frailty prediction in heart failure patients with acute infections: the potential role of thiazide diuretics?
Source: PLoS One. 2026 Aug 12;21(8):e0355848. doi: 10.1371/journal.pone.0355848 (PMC13465814; doi:10.1371/journal.pone.0355848)
Supplement: Peer review history

# Peer review: Decision Letter 0

**DOI:** 10.1371/journal.pone.0355848.r001

**Authors:** Francesco Curcio

6 Jul 2026

PONE-D-26-19524

Frailty prediction in heart failure patients with acute infections: the potential role of thiazide diuretics?

PLOS One?

Dear Dr. Shen,

Thank you for submitting your manuscript to PLOS ONE. After careful consideration, we feel that it has merit but does not fully meet PLOS ONE’s publication criteria as it currently stands. Therefore, we invite you to submit a revised version of the manuscript that addresses the points raised during the review process.

Please submit your revised manuscript by Aug 20 2026 11:59PM. If you will need more time than this to complete your revisions, please reply to this message or contact the journal office at plosone@plos.org. When you're ready to submit your revision, log on to https://www.editorialmanager.com/pone/ and select the 'Submissions Needing Revision' folder to locate your manuscript file.

- A letter that responds to each point raised by the academic editor and reviewer(s). You should upload this letter as a separate file labeled 'Response to Reviewers'.
- A marked-up copy of your manuscript that highlights changes made to the original version. You should upload this as a separate file labeled 'Revised Manuscript with Track Changes'.
- An unmarked version of your revised paper without tracked changes. You should upload this as a separate file labeled 'Manuscript'.

If you would like to make changes to your financial disclosure, please include your updated statement in your cover letter. Guidelines for resubmitting your figure files are available below the reviewer comments at the end of this letter.

If applicable, we recommend that you deposit your laboratory protocols in protocols.io to enhance the reproducibility of your results. Protocols.io assigns your protocol its own identifier (DOI) so that it can be cited independently in the future. For instructions see: https://journals.plos.org/plosone/s/submission-guidelines#loc-laboratory-protocols. Additionally, PLOS ONE offers an option for publishing peer-reviewed Lab Protocol articles, which describe protocols hosted on protocols.io. Read more information on sharing protocols at https://plos.org/protocols?utm_medium=editorial-email&utm_source=authorletters&utm_campaign=protocols.

As the corresponding author, your ORCID iD is verified in the submission system and will appear in the published article. PLOS supports the use of ORCID, and we encourage all coauthors to register for an ORCID iD and use it as well. Please encourage your coauthors to verify their ORCID iD within the submission system before final acceptance, as unverified ORCID iDs will not appear in the published article. *Only*  the individual author can complete the verification step; PLOS staff *cannot*  verify ORCID iDs on behalf of authors.

We look forward to receiving your revised manuscript.

Kind regards,

Francesco Curcio, M.D., Ph.D.

Academic Editor

PLOS One

Journal Requirements:

When submitting your revision, we need you to address these additional requirements.

1. Please ensure that your manuscript meets PLOS ONE’s style requirements, including those for file naming. The PLOS ONE style templates can be found at

https://journals.plos.org/plosone/s/file?id=wjVg/PLOSOne_formatting_sample_main_body.pdf and

https://journals.plos.org/plosone/s/file?id=ba62/PLOSOne_formatting_sample_title_authors_affiliations.pdf.

2. Please note that PLOS One has specific guidelines on code sharing for submissions in which author-generated code underpins the findings in the manuscript. In these cases, all author-generated code must be made available without restrictions upon publication of the work. Please review our guidelines at https://journals.plos.org/plosone/s/materials-and-software-sharing#loc-sharing-code and ensure that your code is shared in a way that follows best practice and facilitates reproducibility and reuse.

3. Thank you for stating in your Funding Statement:

“The author(s) declare that financial support was received for the research and/or publication of this article. This work was supported by the National Natural Science Foundation of China [82173899], the Jiangsu Pharmaceutical Association [H202108, A2021024, Q202202, JY202207, Z04JKM2023E040], and the Hospital Management Innovation Research Project of Jiangsu Provincial Hospital Association (JSYGY-3-2023-264)”

Please provide an amended statement that declares *all* the funding or sources of support (whether external or internal to your organization) received during this study, as detailed online in our guide for authors at http://journals.plos.org/plosone/s/submit-now.  Please also include the statement “There was no additional external funding received for this study.” in your updated Funding Statement.

Please include your amended Funding Statement within your cover letter. We will change the online submission form on your behalf.

4. Thank you for stating the following financial disclosure:

“The author(s) declare that financial support was received for the research and/or publication of this article. This work was supported by the National Natural Science Foundation of China [82173899], the Jiangsu Pharmaceutical Association [H202108, A2021024, Q202202, JY202207, Z04JKM2023E040], and the Hospital Management Innovation Research Project of Jiangsu Provincial Hospital Association (JSYGY-3-2023-264)”

Please state what role the funders took in the study.  If the funders had no role, please state: "The funders had no role in study design, data collection and analysis, decision to publish, or preparation of the manuscript."

If this statement is not correct you must amend it as needed.

Please include this amended Role of Funder statement in your cover letter; we will change the online submission form on your behalf.

5. When completing the data availability statement of the submission form, you indicated that you will make your data available on acceptance. We strongly recommend all authors decide on a data sharing plan before acceptance, as the process can be lengthy and hold up publication timelines. Please note that, though access restrictions are acceptable now, your entire data will need to be made freely accessible if your manuscript is accepted for publication. This policy applies to all data except where public deposition would breach compliance with the protocol approved by your research ethics board. If you are unable to adhere to our open data policy, please kindly revise your statement to explain your reasoning and we will seek the editor’s input on an exemption. Please be assured that, once you have provided your new statement, the assessment of your exemption will not hold up the peer review process.

6. PLOS requires an ORCID iD for the corresponding author in Editorial Manager on papers submitted after December 6th, 2016. Please ensure that you have an ORCID iD and that it is validated in Editorial Manager. To do this, go to ‘Update my Information’ (in the upper left-hand corner of the main menu), and click on the Fetch/Validate link next to the ORCID field. This will take you to the ORCID site and allow you to create a new iD or authenticate a pre-existing iD in Editorial Manager.

7. Please be informed that funding information should not appear in the Acknowledgments section or other areas of your manuscript. We will only publish funding information present in the Funding Statement section of the online submission form. Please remove any funding-related text from the manuscript.

8. Your ethics statement should only appear in the Methods section of your manuscript. If your ethics statement is written in any section besides the Methods, please delete it from any other section.

9. We notice that your supplementary tables are included in the manuscript file. Please remove them and upload them with the file type 'Supporting Information'. Please ensure that each Supporting Information file has a legend listed in the manuscript after the references list.

10. If the reviewer comments include a recommendation to cite specific previously published works, please review and evaluate these publications to determine whether they are relevant and should be cited. There is no requirement to cite these works unless the editor has indicated otherwise.

11. Please review your reference list to ensure that it is complete and correct. If you have cited papers that have been retracted, please include the rationale for doing so in the manuscript text, or remove these references and replace them with relevant current references. Any changes to the reference list should be mentioned in the rebuttal letter that accompanies your revised manuscript. If you need to cite a retracted article, indicate the article’s retracted status in the References list and also include a citation and full reference for the retraction notice.

Additional Editor Comments (if provided):

The manuscript is generally well conducted and addresses a clinically relevant question. Most of the requested revisions concern clarification of the methodology, more cautious interpretation of the observational findings, and improvement of the presentation rather than substantial additional analyses. The manuscript would also benefit from careful language editing and a reduction of redundancy throughout the Introduction and Discussion.

[Note: HTML markup is below. Please do not edit.]

Reviewers' comments:

Reviewer’s Responses to Questions

**Comments to the Author**

1. Is the manuscript technically sound, and do the data support the conclusions?

Reviewer #1: Yes

Reviewer #2: Yes

**********

2. Has the statistical analysis been performed appropriately and rigorously?

Reviewer #1: Yes

Reviewer #2: Yes

**********

3. Have the authors made all data underlying the findings in their manuscript fully available?

The PLOS Data policy

Reviewer #1: Yes

Reviewer #2: Yes

**********

4. Is the manuscript presented in an intelligible fashion and written in standard English?

Reviewer #1: Yes

Reviewer #2: Yes

**********

Reviewer #1: Reviewer Report

Manuscript title: Frailty prediction in heart failure patients with acute infections: the potential role of thiazide diuretics?

Journal: PLOS ONE

Manuscript ID: PONE-D-26-19524

Recommendation: Minor Revision

Overall assessment

This manuscript investigates frailty risk factors and develops a machine-learning-based prediction model among older patients hospitalized with heart failure (HF) complicated by acute infections. The authors analyzed a relatively large cohort of 1,498 patients and applied several machine learning approaches, including LASSO-based feature selection, XGBoost modeling, SHAP-based interpretation, and an online prediction tool. The study addresses a clinically relevant topic, as frailty represents an important determinant of prognosis and treatment complexity in older patients with acute cardiovascular conditions.

The manuscript is generally well structured, the methodology is described in sufficient detail, and the use of explainable artificial intelligence approaches represents a strength. The model performance appears promising, with XGBoost achieving good discrimination (AUROC 0.872) and high AUPRC values. However, several points require clarification and refinement before the manuscript can be considered for publication. In particular, the interpretation of thiazide diuretic use as a protective factor against frailty requires more cautious discussion because of the observational design and potential confounding factors.

I recommend minor revision.

________________________________________

Major comments

1. Interpretation of the association between thiazide diuretics and reduced frailty risk

The main novelty of the manuscript is the identification of thiazide diuretic use as an important predictor associated with lower frailty risk. However, the current wording sometimes implies a possible protective effect, which may overstate the findings.

Because this is a retrospective observational study, the association may be influenced by:

• confounding by indication;

• differences in disease severity;

• baseline functional status;

• renal function;

• physician treatment selection;

• concomitant HF therapies.

Patients receiving thiazide diuretics may represent a subgroup with different clinical characteristics compared with untreated patients.

The authors should:

• clearly state that the finding represents an association rather than causation;

• expand the limitations section regarding residual confounding;

• avoid suggesting that thiazide therapy may prevent or reverse frailty without prospective validation.

________________________________________

2. Definition and timing of frailty assessment

Frailty was assessed using the Clinical Frailty Scale (CFS), with frailty defined as CFS ≥5. The manuscript states that assessments were performed after resolution of acute infection and at discharge. This is important but requires additional clarification.

The authors should explain:

• whether CFS reflected pre-admission baseline frailty or acute illness-related functional decline;

• whether the acute infection itself could temporarily worsen functional status;

• whether CFS assessment at discharge may introduce reverse causality, since hospitalization-related decline may influence frailty classification.

A clearer description of the clinical context of CFS evaluation would improve interpretation.

________________________________________

3. Machine learning methodology and model validation

The methodological approach is generally appropriate; however, some aspects require clarification.

The authors should provide more details regarding:

• whether feature selection was performed strictly within the training dataset to avoid information leakage;

• whether preprocessing steps (including KNN imputation and scaling) were performed only on the training set before application to the test set;

• whether hyperparameter optimization was nested within cross-validation.

Given that the dataset originates from a single institution, external validation is necessary before clinical implementation.

The authors should emphasize that the online calculator represents a proof-of-concept tool rather than a clinically ready application.

________________________________________

4. High prevalence of frailty and model calibration

Frailty prevalence was very high (80.3%), which reflects the selected population but may influence model performance metrics. The authors appropriately considered AUROC and AUPRC; however, calibration becomes particularly important in this context.

The manuscript should provide:

• more detailed calibration results;

• discussion of potential impact of prevalence imbalance;

• clarification of whether predicted probabilities are reliable for clinical decision-making.

________________________________________

Minor comments

1. Abstract

The conclusion in the abstract should be moderated. The sentence:

“This study demonstrated that the use of thiazide diuretics was associated with reduced frailty risk”

should be revised to:

“This study identified thiazide diuretic use as an independent predictor associated with lower frailty probability.”

This better reflects the observational design.

________________________________________

2. Introduction

The introduction is comprehensive but could be shortened. Some statements regarding machine learning as a “virtual clinician” are somewhat exaggerated and should be toned down to maintain scientific neutrality.

________________________________________

3. Methods – inclusion criteria

The exclusion of NYHA class I patients should be justified more clearly, as this criterion may introduce selection bias.

________________________________________

4. Missing data handling

The authors used KNN imputation for missing values. Please clarify:

• whether missingness was assumed to be missing completely at random (MCAR), missing at random (MAR), or not assessed;

• whether sensitivity analyses using alternative imputation methods were considered.

________________________________________

5. Feature selection

The manuscript combines univariate analysis followed by LASSO selection. This approach may exclude clinically relevant variables that are not statistically significant individually but contribute to multivariable prediction.

The authors should briefly justify this strategy.

________________________________________

6. SHAP interpretation

The SHAP analysis is a valuable component of the study. However, the authors should clarify whether SHAP values represent:

• global feature importance;

• directionality of association;

• individual patient-level interpretation.

________________________________________

7. Figures and supplementary material

The figures should be checked to ensure that:

• axes and legends are sufficiently explained;

• abbreviations are defined;

• readers without machine learning expertise can interpret the results.

8. Discussion

The discussion should include a clearer comparison with previous frailty prediction models in HF and geriatric populations.

The authors should also discuss whether the identified predictors (albumin, renal function, lymphocyte percentage, functional capacity, age) reflect known biological pathways of frailty rather than disease-specific predictors.

I strongly suggest to see and discuss the following references:

Multidimensional frailty: Testa G et al., Multidimensional frailty evaluation in elderly outpatients with chronic heart failure: A prospective study. Eur J Prev Cardiol. 2019 Jul;26(10):1115-1117.

Low arterial pressure and catabolic syndrome: Curcio F et al. . The reverse metabolic syndrome in the elderly: Is it a "catabolic" syndrome? Aging Clin Exp Res. 2018 Jun;30(6):547-554.

9. Data availability statement

The current data availability statement appears inconsistent with PLOS ONE requirements. Since clinical data were analyzed, the authors should clarify whether anonymized data can be made available through an appropriate repository or upon reasonable request, according to ethical restrictions.

Suggested final decision to Editor

Minor Revision

The manuscript presents an interesting and potentially valuable machine-learning approach for frailty prediction in older patients with heart failure and acute infections. The study has methodological strengths, particularly the use of explainable AI techniques. The main issue requiring revision is the interpretation of the association between thiazide diuretics and frailty, which should be framed more cautiously given the retrospective observational design. Additional clarification regarding methodology, validation, and limitations would improve the manuscript substantially. Some important references should be added to revised form of the manuscript

Reviewer #2: The manuscript has a strong methodological foundation, a large sample size, and is hbased on a clinically relevant question. The main opportunities for improvement are in presentation rather than scientific content. Specifically, I would recommend:

reducing redundancy by approximately 20–30%, improving transitions between paragraphs, shortening overly long explanatory sections, consolidating repeated descriptions of previous studies' limitations, and making the rationale progress more directly from the clinical problem to the need for an interpretable prediction model. I estimate these revisions could reduce the manuscript by 1,000–1,500 words while making it more concise and easier to follow, without sacrificing scientific rigor.

ie Some background paragraphs explain concepts already familiar to most readers.

For example, detailed descriptions of machine learning visualization tumor imaging examples are not essential.

The paragraph discussing tumor imaging can probably be removed entirely because it does not directly support the rationale of this study. Instead, simply state that SHAP improves model interpretability.

**********

what does this mean?). If published, this will include your full peer review and any attached files.

If you choose “no”, your identity will remain anonymous but your review may still be made public.

**Do you want your identity to be public for this peer review?**  For information about this choice, including consent withdrawal, please see our Privacy Policy

Reviewer #1: No

Reviewer #2: No

**********

[NOTE: If reviewer comments were submitted as an attachment file, they will be attached to this email and accessible via the submission site. Please log into your account, locate the manuscript record, and check for the action link "View Attachments". If this link does not appear, there are no attachment files.]

To ensure your figures meet our technical requirements, please review our figure guidelines: https://journals.plos.org/plosone/s/figures

You may also use PLOS’s free figure tool, NAAS, to help you prepare publication quality figures: https://journals.plos.org/plosone/s/figures#loc-tools-for-figure-preparation.

NAAS will assess whether your figures meet our technical requirements by comparing each figure against our figure specifications.

# Author comment: Author response to Decision Letter 1

**DOI:** 10.1371/journal.pone.0355848.r002

20 Jul 2026

Point-by-point responses to the reviewers and editor have been submitted as a file.

Attachment Submitted filename: Response to Reviewers.docx

# Peer review: Decision Letter 1

**DOI:** 10.1371/journal.pone.0355848.r003

**Authors:** Francesco Curcio

27 Jul 2026

Frailty prediction in heart failure patients with acute infections: the potential role of thiazide diuretics?

PONE-D-26-19524R1

Dear Dr. Shen,

We’re pleased to inform you that your manuscript has been judged scientifically suitable for publication and will be formally accepted for publication once it meets all outstanding technical requirements.

Within one week, you’ll receive an e-mail detailing the required amendments. When these have been addressed, you’ll receive a formal acceptance letter and your manuscript will be scheduled for publication.

An invoice will be generated when your article is formally accepted. Please note, if your institution has a publishing partnership with PLOS and your article meets the relevant criteria, all or part of your publication costs will be covered. Please make sure your user information is up-to-date by logging into Editorial Manager at Editorial Manager® and clicking the ‘Update My Information' link at the top of the page. For questions related to billing, please contact billing support.

If your institution or institutions have a press office, please notify them about your upcoming paper to help maximize its impact. If they’ll be preparing press materials, please inform our press team as soon as possible -- no later than 48 hours after receiving the formal acceptance. Your manuscript will remain under strict press embargo until 2 pm Eastern Time on the date of publication. For more information, please contact onepress@plos.org.

Kind regards,

Francesco Curcio, M.D., Ph.D.

Academic Editor

PLOS One

Additional Editor Comments (optional):

According to the reviewers, the manuscript has been substantially improved and now merits publication.

Reviewers' comments:

Reviewer’s Responses to Questions

**Comments to the Author**

Reviewer #1: All comments have been addressed

Reviewer #2: All comments have been addressed

**********

2. Is the manuscript technically sound, and do the data support the conclusions?

Reviewer #1: Yes

Reviewer #2: Yes

**********

3. Has the statistical analysis been performed appropriately and rigorously?

Reviewer #1: Yes

Reviewer #2: Yes

**********

4. Have the authors made all data underlying the findings in their manuscript fully available?

The PLOS Data policy

Reviewer #1: Yes

Reviewer #2: Yes

**********

5. Is the manuscript presented in an intelligible fashion and written in standard English?

Reviewer #1: Yes

Reviewer #2: Yes

**********

Reviewer #1: All questions arised have been answered. No further comments. The manuscript merits to be published in Plos ONE.

Reviewer #2: (No Response)

**********

what does this mean?). If published, this will include your full peer review and any attached files.

If you choose “no”, your identity will remain anonymous but your review may still be made public.

**Do you want your identity to be public for this peer review?**  For information about this choice, including consent withdrawal, please see our Privacy Policy

Reviewer #1: No

Reviewer #2: No

**********

# Editor report: Acceptance letter

**DOI:** 10.1371/journal.pone.0355848.r004

**Authors:** Francesco Curcio

PONE-D-26-19524R1

PLOS One

Dear Dr. Shen,

I'm pleased to inform you that your manuscript has been deemed suitable for publication in PLOS One. Congratulations! Your manuscript is now being handed over to our production team.

At this stage, our production department will prepare your paper for publication. This includes ensuring the following:

* All references, tables, and figures are properly cited

* All relevant supporting information is included in the manuscript submission,

* There are no issues that prevent the paper from being properly typeset

You will receive further instructions from the production team, including instructions on how to review your proof when it is ready. Please keep in mind that we are working through a large volume of accepted articles, so please give us a few days to review your paper and let you know the next and final steps.

Lastly, if your institution or institutions have a press office, please let them know about your upcoming paper now to help maximize its impact. If they'll be preparing press materials, please inform our press team within the next 48 hours. Your manuscript will remain under strict press embargo until 2 pm Eastern Time on the date of publication. For more information, please contact onepress@plos.org.

You will receive an invoice from PLOS for your publication fee after your manuscript has reached the completed accept phase. If you receive an email requesting payment before acceptance or for any other service, this may be a phishing scheme. Learn how to identify phishing emails and protect your accounts at https://explore.plos.org/phishing.

If we can help with anything else, please email us at customercare@plos.org.

Thank you for submitting your work to PLOS ONE and supporting open access.

Kind regards,

PLOS ONE Editorial Office Staff

on behalf of

Dr. Francesco Curcio

Academic Editor

PLOS One
